# Lycopene overproduction in *Saccharomyces cerevisiae* through combining pathway engineering with host engineering

**DOI:** 10.1186/s12934-016-0509-4

**Published:** 2016-06-21

**Authors:** Yan Chen, Wenhai Xiao, Ying Wang, Hong Liu, Xia Li, Yingjin Yuan

**Affiliations:** Key Laboratory of Systems Bioengineering (Ministry of Education), Tianjin University, Tianjin, 300072 People’s Republic of China; SynBio Research Platform, Collaborative Innovation Center of Chemical Science and Engineering (Tianjin), School of Chemical Engineering and Technology, Tianjin University, Tianjin, 300072 People’s Republic of China

**Keywords:** Metabolic engineering, Lycopene, *Saccharomyces cerevisiae*, Heterologous pathway, Synthetic biology

## Abstract

**Background:**

Microbial production of lycopene, a commercially and medically important compound, has received increasing concern in recent years. *Saccharomyces cerevisiae* is regarded as a safer host for lycopene production than *Escherichia coli*. However, to date, the lycopene yield (mg/g DCW) in *S. cerevisiae* was lower than that in *E. coli* and did not facilitate downstream extraction process, which might be attributed to the incompatibility between host cell and heterologous pathway. Therefore, to achieve lycopene overproduction in *S. cerevisiae*, both host cell and heterologous pathway should be delicately engineered.

**Results:**

In this study, lycopene biosynthesis pathway was constructed by integration of *CrtE*, *CrtB* and *CrtI* in *S. cerevisiae* CEN.PK2. When *YPL062W*, a distant genetic locus, was deleted, little acetate was accumulated and approximately 100 % increase in cytosolic acetyl-CoA pool was achieved relative to that in parental strain. Through screening CrtE, CrtB and CrtI from diverse species, an optimal carotenogenic enzyme combination was obtained, and CrtI from *Blakeslea trispora* (BtCrtI) was found to have excellent performance on lycopene production as well as lycopene proportion in carotenoid. Then, the expression level of Bt*CrtI* was fine-tuned and the effect of cell mating types was also evaluated. Finally, potential distant genetic targets (*YJL064W*, *ROX1*, and *DOS2*) were deleted and a stress-responsive transcription factor *INO2* was also up-regulated. Through the above modifications between host cell and carotenogenic pathway, lycopene yield was increased by approximately 22-fold (from 2.43 to 54.63 mg/g DCW). Eventually, in fed-batch fermentation, lycopene production reached 55.56 mg/g DCW, which is the highest reported yield in yeasts.

**Conclusions:**

*Saccharomyces cerevisiae* was engineered to produce lycopene in this study. Through combining host engineering (distant genetic loci and cell mating types) with pathway engineering (enzyme screening and gene fine-tuning), lycopene yield was stepwise improved by 22-fold as compared to the starting strain. The highest lycopene yield (55.56 mg/g DCW) in yeasts was achieved in 5-L bioreactors. This study provides a good reference of combinatorial engineering of host cell and heterologous pathway for microbial overproduction of pharmaceutical and chemical products.

**Electronic supplementary material:**

The online version of this article (doi:10.1186/s12934-016-0509-4) contains supplementary material, which is available to authorized users.

## Background

Artificial biosynthetic pathway and host cell are two fundamental elements for microbe-based heterologous biosynthesis of natural products. On one hand, potential metabolic and regulatory issues from host cell play an important role in pathway productivity [1–4]. One-hundred distant genetic loci that are not directly involved in target pathway were identified to influence carotenoid production significantly in *Saccharomycescerevisiae* [5]. On the other hand, balanced metabolic flux between modules in target pathway is another important issue to improve pathway performance. Through multivariate-modular optimization of taxadiene metabolic pathway, a 15,000-fold increase in taxadiene titer was observed in *Escherichia coli* [6]. A “push–pull-block” pathway manipulation strategy significantly enhanced terpenoids production in yeasts [7, 8]. Thus, optimal pathway output can be achieved by means of delicate engineering of both target pathway and host cell [9]. It was reported that bisabolene production in *S. cerevisiae* was increased by 20 times through deleting multiple distant genes related to intracellular mevalonate level and manipulating the expression level of three genes involved in mevalonate (MVA) pathway [10]. Swidah et al. [11] reported that through the combinatorial effects of deletion of *ADH1* to restore redox imbalance, expression of a butanol resistant allele *GCD1*, and manipulation of acetyl-CoA formation module, butanol production in *S. cerevisiae* was increased by more than 30 times. In a word, combinatorial engineering host cell with heterologous pathway offers a promising alternative to achieve better metabolic flux balance and higher output of heterologous pathway.

Lycopene has long been used as functional food, nutraceutical, pharmaceutical and cosmetic due to its anti-oxidative and anti-cancer activities [12, 13]. Compared to chemical synthesis and extraction from tomatoes, microbial production of lycopene is more economical and sustainable. In recent years, lycopene production was successfully realized in *Blakeslea trispora*, *E. coli* and yeasts. However, regarding to food safety issues, it is controversial to use *B. trispora* or *E. coli* for lycopene synthesis, since *E. coli* would release endotoxin [14] and *B. trispora* requires the addition of cyclase inhibitors [15]. *Saccharomyces cerevisiae* is generally recognized as safe (GRAS), robust and preferred organism for industrial use. To date, lycopene yield in *S. cerevisiae* was increased to 24.41 mg/g DCW with elaborate efforts in directed evolution and copy number variation of *Crt* genes from *Xanthophyllomyces dendrorhous* [16]. However, the lycopene yield was still much lower than that in *E. coli* [17, 18], which did not facilitate downstream extraction process. It was speculated that such low yield might be attributed to the incompatibility between *S. cerevisiae* and the heterologous pathway. Therefore, combinatorial engineering *S. cerevisiae* with a heterologous pathway may offer an effective solution to enhance lycopene yield.

**Fig. 1:**
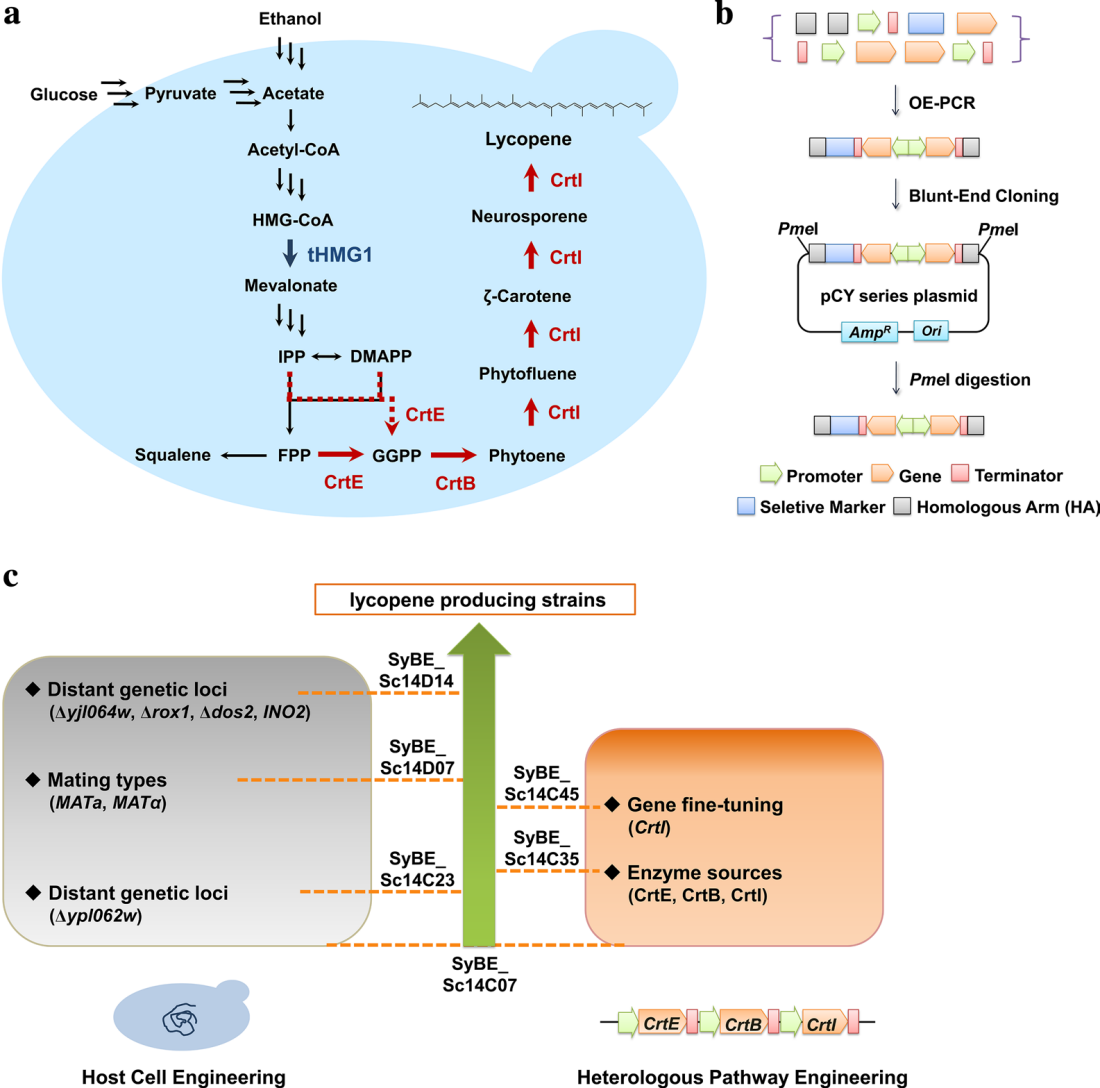
Schematic representation of the engineering strategies for enhanced lycopene production in *S. cerevisiae*. **a** The engineered lycopene biosynthetic pathway in *S. cerevisiae*. Genetic modifications are noted by *thick arrows*. *Red arrows* indicate the heterologous lycopene biosynthetic pathway consisting of CrtE, CrtB and CrtI, which starts either from FPP (*solid arrow*) or directly from IPP and DMAPP (*dashed arrow*). The enzyme overexpressed in the described lycopene-producing strain is highlighted in *blue*. **b** Construction of plasmids and integration modules. **c** The rationale of experiment design: combinatorial engineering of host cell and heterologous pathway

In this study, heterologous carotenogenic pathway and its recruited host *S. cerevisiae* were combinatorially engineered (Fig. 1c). Acetyl-CoA formation was enhanced by the deletion of *YPL062W*. A novel and optimal combination of geranylgeranyl diphosphate synthase (CrtE, or GGPPS), phytoene synthase (CrtB) and phytoene desaturase (CrtI) was generated through related enzyme screening from diverse species. The expression level of *CrtI*, which has significant impact on lycopene production as well as lycopene proportion in carotenoid, was fine-tuned by varying promoter strength and copy number. The influences of different cell mating types and potential distant genetic loci were also evaluated. Using this combinatorial engineering strategy, we achieved approximately 22-fold improvement (up to 54.63 mg/g DCW) in lycopene yield, which provides a good reference to increase the compatibility between heterologous pathway and host cell for microbial production of valuable molecules.

## Methods

### Materials

All oligonucleotides were purchased from Invitrogen. Q5 DNA polymerase and *Pme*I restriction endonuclease were purchased from New England Biolabs (MA, USA). DNA purification and plasmids isolation kits were purchased from Tiangen (Beijing, China). CloneJET PCR Cloning Kit was purchased from Fermentas (MD, USA). DNA sequencing was conducted by Genewiz (Beijing, China). Standards of lycopene and phytoene were purchased from Sigma (Sigma-Aldrich, MO, USA). Standards of phytofluene, ζ-carotene and neurosporene were purchased from Express (Beijing, China).

### Strains and culture conditions

*Escherichia coli* DH5α was used for routine cloning procedures, and was cultivated at 37 °C in Luria–Bertani (LB) medium containing 100 μg/mL ampicillin for selection. All the yeast strains engineered in this study are based on homologous haploid *S. cerevisiae* strains, CEN.PK2-1C (*MATa*) or CEN.PK2-1D (*MATα*). Engineered yeast strains were selected on synthetic complete (SC) medium (0.67 % yeast nitrogen base without amino acids, 2 % glucose, and appropriate amino acid drop-out mix), or YPD medium (2 % peptone, 1 % yeast extract, and 2 % glucose) with 200 μg/mL geneticin, 300 μg/mL hygromycin B or 50 μg/mL bleomycin when needed. YPDG medium, consisting of 2 % glucose (unless otherwise indicated), 2 % peptone, 1 % yeast extract and 1 % D-(+)-galactose, was used for shake-flask fermentations.

For shake-flask fermentation, yeast glycerol-stock was inoculated into a tube containing 2 mL YPD medium for overnight growth, then all the preculture was inoculated into a 250 mL shake-flask containing 25 mL YPD. After growing to the mid-log phase, the seed was transferred to 50 mL fresh YPDG medium at an initial OD_600_ of 0.5 and grown at 30 °C for 48 h.

### Construction of plasmids and strains

*Saccharomyces cerevisiae* strains and plasmids used in this study are summarized in Table 1. All oligonucleotides used for construction of the above plasmids and strains are listed in Additional file 1: Table S1. Construction procedures of plasmids and integration modules are shown in Fig. 1b. All heterologous genes used for lycopene biosynthesis were codon-optimized and synthesized by Genewiz (Beijing, China) for expression in *S. cerevisiae*. Endogenous truncated 3-hydroxy-3-methylglutaryl coenzyme A reductase (*tHMG1*) [19], promoters, terminators and integration homologous arms (except *TRP1* homologous arm) used in this study were amplified from the genomic DNA of *S. cerevisiae* CEN.PK2-1C. Auxotroph markers (*LEU2*, *HIS3*) and *TRP1* homologous arm were amplified from the genomic DNA of *S. cerevisiae* S288C. Antibiotic markers (*KanMX*, *HphMX* and *BleMX*) were amplified from plasmid pKan, pHph and pBle (owned by our laboratory), respectively. Overlap extension PCR (OE-PCR) was used to assemble the above parts into modules according to Additional file 1: Figure S1. The resulting modules were purified and cloned into pJET1.2/blunt vector following the protocol of CloneJET PCR Cloning Kit (Fermentas, MD, USA), obtaining plasmid pCY series (Table 1). Finally, all the integration modules were digested from plasmids with *Pme*I, then purified and transformed into yeast for genomic integration using the LiAc/SS carrier DNA/PEG method [20]. For gene deletions, one-step integration of PCR-amplified deletion cassettes was adopted [21]. Gene deletions and genomic integrations were verified by diagnostic PCR.

**Table 1:** *S. cerevisiae* strains and plasmids used in this study

	Description	Source
Strain name		
CEN.PK2-1C	*MATa*, *ura3*-*52*, *trp1*-*289*, *leu2*-*3,112*, *his3Δ1*, *MAL2*-*8C*, *SUC2*	EUROSCARF
CEN.PK2-1D	*MATα*, *ura3*-*52*, *trp1*-*289*, *leu2*-*3,112*, *his3Δ1*, *MAL2*-*8C*, *SUC2*	EUROSCARF
SyBE_Sc14C01	CEN.PK2-1C, Δ*gal80::HIS3*	This study
SyBE_Sc14C02	CEN.PK2-1C, Δ*gal1* Δ*gal7* Δ*gal10::HIS3*	This study
SyBE_Sc14C06	SyBE_Sc14C01, *trp1::TRP1_*T_*CYC1*_-Bt*CrtI*-P_*GAL10*_-P_*GAL1*_-Pa*CrtB*-T_*PGK1*_, *leu2::LEU2_*T_*ACT1*_-*tHMG1*-P_*GAL10*_-P_*GAL1*_-Pa*CrtE*-T_*GPM1*_	This study
SyBE_Sc14C07	SyBE_Sc14C02, *trp1::TRP1_*T_*CYC1*_-Bt*CrtI*-P_*GAL10*_-P_*GAL1*_-Pa*CrtB*-T_*PGK1*_, *leu2::LEU2_*T_*ACT1*_-*tHMG1*-P_*GAL10*_-P_*GAL1*_-Pa*CrtE*-T_*GPM1*_	This study
SyBE_Sc14C10	CEN.PK2-1C, Δ*gal1* Δ*gal7* Δ*gal10::HIS3*, Δ*ypl062w::KanMX*	This study
SyBE_Sc14C21	SyBE_Sc14C10, *trp1::TRP1_*T_*CYC1*_-Aa*CrtI*-P_*GAL10*_-P_*GAL1*_-Aa*CrtB*-T_*PGK1*_, *leu2::LEU2_*T_*ACT1*_-*tHMG1*-P_*GAL10*_-P_*GAL1*_-Pa*CrtE*-T_*GPM1*_	This study
SyBE_Sc14C51	SyBE_Sc14C10, *trp1::TRP1_*T_*CYC1*_-Pa*CrtI*-P_*GAL10*_-P_*GAL1*_-Aa*CrtB*-T_*PGK1*_, *leu2::LEU2_*T_*ACT1*_-*tHMG1*-P_*GAL10*_-P_*GAL1*_-Pa*CrtE*-T_*GPM1*_	This study
SyBE_Sc14C52	SyBE_Sc14C10, *trp1::TRP1_*T_*CYC1*_-Bt*CrtI*-P_*GAL10*_-P_*GAL1*_-Aa*CrtB*-T_*PGK1*_, *leu2::LEU2_*T_*ACT1*_-*tHMG1*-P_*GAL10*_-P_*GAL1*_-Pa*CrtE*-T_*GPM1*_	This study
SyBE_Sc14C53	SyBE_Sc14C10, *trp1::TRP1_*T_*CYC1*_-Aa*CrtI*-P_*GAL10*_-P_*GAL1*_-Pa*CrtB*-T_*PGK1*_, *leu2::LEU2_*T_*ACT1*_-*tHMG1*-P_*GAL10*_-P_*GAL1*_-Pa*CrtE*-T_*GPM1*_	This study
SyBE_Sc14C22	SyBE_Sc14C10, *trp1::TRP1_*T_*CYC1*_-Pa*CrtI*-P_*GAL10*_-P_*GAL1*_-Pa*CrtB*-T_*PGK1*_, *leu2::LEU2_*T_*ACT1*_-*tHMG1*-P_*GAL10*_-P_*GAL1*_-Pa*CrtE*-T_*GPM1*_	This study
SyBE_Sc14C23	SyBE_Sc14C10, *trp1::TRP1_*T_*CYC1*_-Bt*CrtI*-P_*GAL10*_-P_*GAL1*_-Pa*CrtB*-T_*PGK1*_, *leu2::LEU2_*T_*ACT1*_-*tHMG1*-P_*GAL10*_-P_*GAL1*_-Pa*CrtE*-T_*GPM1*_	This study
SyBE_Sc14C24	SyBE_Sc14C10, *trp1::TRP1_*T_*CYC1*_-Aa*CrtI*-P_*GAL10*_-P_*GAL1*_-Aa*CrtB*-T_*PGK1*_, *leu2::LEU2_*T_*ACT1*_-*tHMG1*-P_*GAL10*_-P_*GAL1*_-Sa*CrtE*-T_*GPM1*_	This study
SyBE_Sc14C54	SyBE_Sc14C10, *trp1::TRP1_*T_*CYC1*_-Pa*CrtI*-P_*GAL10*_-P_*GAL1*_-Aa*CrtB*-T_*PGK1*_, *leu2::LEU2_*T_*ACT1*_-*tHMG1*-P_*GAL10*_-P_*GAL1*_-Sa*CrtE*-T_*GPM1*_	This study
SyBE_Sc14C55	SyBE_Sc14C10, *trp1::TRP1_*T_*CYC1*_-Bt*CrtI*-P_*GAL10*_-P_*GAL1*_-Aa*CrtB*-T_*PGK1*_, *leu2::LEU2_*T_*ACT1*_-*tHMG1*-P_*GAL10*_-P_*GAL1*_-Sa*CrtE*-T_*GPM1*_	This study
SyBE_Sc14C56	SyBE_Sc14C10, *trp1::TRP1_*T_*CYC1*_-Aa*CrtI*-P_*GAL10*_-P_*GAL1*_-Pa*CrtB*-T_*PGK1*_, *leu2::LEU2_*T_*ACT1*_-*tHMG1*-P_*GAL10*_-P_*GAL1*_-Sa*CrtE*-T_*GPM1*_	This study
SyBE_Sc14C25	SyBE_Sc14C10, *trp1::TRP1_*T_*CYC1*_-Pa*CrtI*-P_*GAL10*_-P_*GAL1*_-Pa*CrtB*-T_*PGK1*_, *leu2::LEU2_*T_*ACT1*_-*tHMG1*-P_*GAL10*_-P_*GAL1*_-Sa*CrtE*-T_*GPM1*_	This study
SyBE_Sc14C26	SyBE_Sc14C10, *trp1::TRP1_*T_*CYC1*_-Bt*CrtI*-P_*GAL10*_-P_*GAL1*_-Pa*CrtB*-T_*PGK1*_, *leu2::LEU2_*T_*ACT1*_-*tHMG1*-P_*GAL10*_-P_*GAL1*_-Sa*CrtE*-T_*GPM1*_	This study
SyBE_Sc14C27	SyBE_Sc14C10, *trp1::TRP1_*T_*CYC1*_-Aa*CrtI*-P_*GAL10*_-P_*GAL1*_-Aa*CrtB*-T_*PGK1*_, *leu2::LEU2_*T_*ACT1*_-*tHMG1*-P_*GAL10*_-P_*GAL1*_-Af*CrtE*-T_*GPM1*_	This study
SyBE_Sc14C57	SyBE_Sc14C10, *trp1::TRP1_*T_*CYC1*_-Pa*CrtI*-P_*GAL10*_-P_*GAL1*_-Aa*CrtB*-T_*PGK1*_, *leu2::LEU2_*T_*ACT1*_-*tHMG1*-P_*GAL10*_-P_*GAL1*_-Af*CrtE*-T_*GPM1*_	This study
SyBE_Sc14C58	SyBE_Sc14C10, *trp1::TRP1_*T_*CYC1*_-Bt*CrtI*-P_*GAL10*_-P_*GAL1*_-Aa*CrtB*-T_*PGK1*_, *leu2::LEU2_*T_*ACT1*_-*tHMG1*-P_*GAL10*_-P_*GAL1*_-Af*CrtE*-T_*GPM1*_	This study
SyBE_Sc14C59	SyBE_Sc14C10, *trp1::TRP1_*T_*CYC1*_-Aa*CrtI*-P_*GAL10*_-P_*GAL1*_-Pa*CrtB*-T_*PGK1*_, *leu2::LEU2_*T_*ACT1*_-*tHMG1*-P_*GAL10*_-P_*GAL1*_-Af*CrtE*-T_*GPM1*_	This study
SyBE_Sc14C28	SyBE_Sc14C10, *trp1::TRP1_*T_*CYC1*_-Pa*CrtI*-P_*GAL10*_-P_*GAL1*_-Pa*CrtB*-T_*PGK1*_, *leu2::LEU2_*T_*ACT1*_-*tHMG1*-P_*GAL10*_-P_*GAL1*_-Af*CrtE*-T_*GPM1*_	This study
SyBE_Sc14C29	SyBE_Sc14C10, *trp1::TRP1_*T_*CYC1*_-Bt*CrtI*-P_*GAL10*_-P_*GAL1*_-Pa*CrtB*-T_*PGK1*_, *leu2::LEU2_*T_*ACT1*_-*tHMG1*-P_*GAL10*_-P_*GAL1*_-Af*CrtE*-T_*GPM1*_	This study
SyBE_Sc14C30	SyBE_Sc14C10, *trp1::TRP1_*T_*CYC1*_-Aa*CrtI*-P_*GAL10*_-P_*GAL1*_-Aa*CrtB*-T_*PGK1*_, *leu2::LEU2_*T_*ACT1*_-*tHMG1*-P_*GAL10*_-P_*GAL1*_-Bt*CrtE*-T_*GPM1*_	This study
SyBE_Sc14C60	SyBE_Sc14C10, *trp1::TRP1_*T_*CYC1*_-Pa*CrtI*-P_*GAL10*_-P_*GAL1*_-Aa*CrtB*-T_*PGK1*_, *leu2::LEU2_*T_*ACT1*_-*tHMG1*-P_*GAL10*_-P_*GAL1*_-Bt*CrtE*-T_*GPM1*_	This study
SyBE_Sc14C61	SyBE_Sc14C10, *trp1::TRP1_*T_*CYC1*_-Bt*CrtI*-P_*GAL10*_-P_*GAL1*_-Aa*CrtB*-T_*PGK1*_, *leu2::LEU2_*T_*ACT1*_-*tHMG1*-P_*GAL10*_-P_*GAL1*_-Bt*CrtE*-T_*GPM1*_	This study
SyBE_Sc14C62	SyBE_Sc14C10, *trp1::TRP1_*T_*CYC1*_-Aa*CrtI*-P_*GAL10*_-P_*GAL1*_-Pa*CrtB*-T_*PGK1*_, *leu2::LEU2_*T_*ACT1*_-*tHMG1*-P_*GAL10*_-P_*GAL1*_-Bt*CrtE*-T_*GPM1*_	This study
SyBE_Sc14C31	SyBE_Sc14C10, *trp1::TRP1_*T_*CYC1*_-Pa*CrtI*-P_*GAL10*_-P_*GAL1*_-Pa*CrtB*-T_*PGK1*_, *leu2::LEU2_*T_*ACT1*_-*tHMG1*-P_*GAL10*_-P_*GAL1*_-Bt*CrtE*-T_*GPM1*_	This study
SyBE_Sc14C32	SyBE_Sc14C10, *trp1::TRP1_*T_*CYC1*_-Bt*CrtI*-P_*GAL10*_-P_*GAL1*_-Pa*CrtB*-T_*PGK1*_, *leu2::LEU2_*T_*ACT1*_-*tHMG1*-P_*GAL10*_-P_*GAL1*_-Bt*CrtE*-T_*GPM1*_	This study
SyBE_Sc14C33	SyBE_Sc14C10, *trp1::TRP1_*T_*CYC1*_-Aa*CrtI*-P_*GAL10*_-P_*GAL1*_-Aa*CrtB*-T_*PGK1*_, *leu2::LEU2_*T_*ACT1*_-*tHMG1*-P_*GAL10*_-P_*GAL1*_-Tm*CrtE*-T_*GPM1*_	This study
SyBE_Sc14C63	SyBE_Sc14C10, *trp1::TRP1_*T_*CYC1*_-Pa*CrtI*-P_*GAL10*_-P_*GAL1*_-Aa*CrtB*-T_*PGK1*_, *leu2::LEU2_*T_*ACT1*_-*tHMG1*-P_*GAL10*_-P_*GAL1*_-Tm*CrtE*-T_*GPM1*_	This study
SyBE_Sc14C64	SyBE_Sc14C10, *trp1::TRP1_*T_*CYC1*_-Bt*CrtI*-P_*GAL10*_-P_*GAL1*_-Aa*CrtB*-T_*PGK1*_, *leu2::LEU2_*T_*ACT1*_-*tHMG1*-P_*GAL10*_-P_*GAL1*_-Tm*CrtE*-T_*GPM1*_	This study
SyBE_Sc14C65	SyBE_Sc14C10, *trp1::TRP1_*T_*CYC1*_-Aa*CrtI*-P_*GAL10*_-P_*GAL1*_-Pa*CrtB*-T_*PGK1*_, *leu2::LEU2_*T_*ACT1*_-*tHMG1*-P_*GAL10*_-P_*GAL1*_-Tm*CrtE*-T_*GPM1*_	This study
SyBE_Sc14C34	SyBE_Sc14C10, *trp1::TRP1_*T_*CYC1*_-Pa*CrtI*-P_*GAL10*_-P_*GAL1*_-Pa*CrtB*-T_*PGK1*_, *leu2::LEU2_*T_*ACT1*_-*tHMG1*-P_*GAL10*_-P_*GAL1*_-Tm*CrtE*-T_*GPM1*_	This study
SyBE_Sc14C35	SyBE_Sc14C10, *trp1::TRP1_*T_*CYC1*_-Bt*CrtI*-P_*GAL10*_-P_*GAL1*_-Pa*CrtB*-T_*PGK1*_, *leu2::LEU2_*T_*ACT1*_-*tHMG1*-P_*GAL10*_-P_*GAL1*_-Tm*CrtE*-T_*GPM1*_	This study
SyBE_Sc14C40	SyBE_Sc14C10, *leu2::LEU2*_T_*CYC1*_-*RFP*	This study
SyBE_Sc14C41	SyBE_Sc14C10, *leu2::LEU2*_T_*CYC1*_-*RFP*-P_*GAL3*_	This study
SyBE_Sc14C42	SyBE_Sc14C10, *leu2::LEU2*_T_*CYC1*_-*RFP*-P_*GAL7*_	This study
SyBE_Sc14C43	SyBE_Sc14C10, *leu2::LEU2*_T_*CYC1*_-*RFP*-P_*GAL10*_	This study
SyBE_Sc14C44	SyBE_Sc14C10, *trp1::TRP1_*T_*CYC1*_-Bt*CrtI*-P_*GAL10*_-P_*GAL1*_-Pa*CrtB*-T_*PGK1*_, *leu2::LEU2_*T_*CYC1*_-Bt*CrtI*-P_*GAL3*_-T_*ACT1*_-*tHMG1*-P_*GAL10*_-P_*GAL1*_-Tm*CrtE*-T_*GPM1*_	This study
SyBE_Sc14C45	SyBE_Sc14C10, *trp1::TRP1_*T_*CYC1*_-Bt*CrtI*-P_*GAL10*_-P_*GAL1*_-Pa*CrtB*-T_*PGK1*_, *leu2::LEU2_*T_*CYC1*_-Bt*CrtI*-P_*GAL7*_-T_*ACT1*_-*tHMG1*-P_*GAL10*_-P_*GAL1*_-Tm*CrtE*-T_*GPM1*_	This study
SyBE_Sc14C46	SyBE_Sc14C10, *trp1::TRP1_*T_*CYC1*_-Bt*CrtI*-P_*GAL10*_-P_*GAL1*_-Pa*CrtB*-T_*PGK1*_, *leu2::LEU2_*T_*CYC1*_-Bt*CrtI*-P_*GAL7*_-T_*ACT1*_-*tHMG1*-P_*GAL10*_-P_*GAL1*_-Tm*CrtE*-T_*GPM1*_, Δ*YGLCtau3::HphMX_*P_*GAL7*_-Bt*CrtI*-T_*CYC1*_	This study
SyBE_Sc14D04	CEN.PK2-1D, Δ*gal1* Δ*gal7* Δ*gal10::HIS3*, Δ*ypl062w::KanMX*, *trp1::TRP1_*T_*CYC1*_-Bt*CrtI*-P_*GAL10*_-P_*GAL1*_-Pa*CrtB*-T_*PGK1*_	This study
SyBE_Sc14D05	SyBE_Sc14D04, *leu2::LEU2_*T_*ACT1*_-*tHMG1*-P_*GAL10*_-P_*GAL1*_-Tm*CrtE*-T_*GPM1*_	This study
SyBE_Sc14D06	SyBE_Sc14D04, *leu2::LEU2_*T_*CYC1*_-Bt*CrtI*-P_*GAL3*_-T_*ACT1*_-*tHMG1*-P_*GAL10*_-P_*GAL1*_-Tm*CrtE*-T_*GPM1*_	This study
SyBE_Sc14D07	SyBE_Sc14D04, *leu2::LEU2_*T_*CYC1*_-Bt*CrtI*-P_*GAL7*_-T_*ACT1*_-*tHMG1*-P_*GAL10*_-P_*GAL1*_-Tm*CrtE*-T_*GPM1*_	This study
SyBE_Sc14D08	SyBE_Sc14D04, *leu2::LEU2_*T_*CYC1*_-Bt*CrtI*-P_*GAL7*_-T_*ACT1*_-*tHMG1*-P_*GAL10*_-P_*GAL1*_-Tm*CrtE*-T_*GPM1*_, Δ*YGLCtau3::HphMX_*P_*GAL7*_-Bt*CrtI*-T_*CYC1*_	This study
SyBE_Sc14D10	SyBE_Sc14D04, *leu2::LEU2_*T_*CYC1*_-Bt*CrtI*-P_*GAL7*_-T_*ACT1*_-*tHMG1*-P_*GAL10*_-P_*GAL1*_-Tm*CrtE*-T_*GPM1*_, Δ*yjl064w::BleMX*	This study
SyBE_Sc14D11	SyBE_Sc14D04, *leu2::LEU2_*T_*CYC1*_-Bt*CrtI*-P_*GAL7*_-T_*ACT1*_-*tHMG1*-P_*GAL10*_-P_*GAL1*_-Tm*CrtE*-T_*GPM1*_, Δ*rox1::BleMX*	This study
SyBE_Sc14D12	SyBE_Sc14D04, *leu2::LEU2_*T_*CYC1*_-Bt*CrtI*-P_*GAL7*_-T_*ACT1*_-*tHMG1*-P_*GAL10*_-P_*GAL1*_-Tm*CrtE*-T_*GPM1*_, Δ*dos2::BleMX*	This study
SyBE_Sc14D13	SyBE_Sc14D04, *leu2::LEU2_*T_*CYC1*_-Bt*CrtI*-P_*GAL7*_-T_*ACT1*_-*tHMG1*-P_*GAL10*_-P_*GAL1*_-Tm*CrtE*-T_*GPM1*_, Δ*rox1::BleMX*, Δ*dos2::HphMX*	This study
SyBE_Sc14D14	SyBE_Sc14D04, *leu2::LEU2_*T_*CYC1*_-Bt*CrtI*-P_*GAL7*_-T_*ACT1*_-*tHMG1*-P_*GAL10*_-P_*GAL1*_-Tm*CrtE*-T_*GPM1*_, Δ*rox1::BleMX*, Δ*YPRCdelta15::HphMX_*P_*GAL1*_-*INO2*-T_*CPS1*_	This study
Plasmid		
pJET1.2/blunt	Blunt-end PCR fragments cloning vector	Fermentas
pCY01	pJET1.2/blunt possessing *TRP1* homologous arm, T_*CYC1*_-Aa*CrtI*-P_*GAL10*_-P_*GAL1*_-Aa*CrtB*-T_*PGK1*_	This study
pCY02	pJET1.2/blunt possessing *TRP1* homologous arm, T_*CYC1*_-Pa*CrtI*-P_*GAL10*_-P_*GAL1*_-Pa*CrtB*-T_*PGK1*_	This study
pCY03	pJET1.2/blunt possessing *TRP1* homologous arm, T_*CYC1*_-Bt*CrtI*-P_*GAL10*_-P_*GAL1*_-Pa*CrtB*-T_*PGK1*_	This study
pCY04	pJET1.2/blunt possessing *LEU2* homologous arm with *LEU2* marker, T_*ACT1*_-*tHMG1*-P_*GAL10*_-P_*GAL1*_-Pa*CrtE*-T_*GPM1*_	This study
pCY05	pJET1.2/blunt possessing *LEU2* homologous arm with *LEU2* marker, T_*ACT1*_-*tHMG1*-P_*GAL10*_-P_*GAL1*_-Sa*CrtE*-T_*GPM1*_	This study
pCY06	pJET1.2/blunt possessing *LEU2* homologous arm with *LEU2* marker, T_*ACT1*_-*tHMG1*-P_*GAL10*_-P_*GAL1*_-Af*CrtE*-T_*GPM1*_	This study
pCY07	pJET1.2/blunt possessing *LEU2* homologous arm with *LEU2* marker, T_*ACT1*_-*tHMG1*-P_*GAL10*_-P_*GAL1*_-Bt*CrtE*-T_*GPM1*_	This study
pCY08	pJET1.2/blunt possessing *LEU2* homologous arm with *LEU2* marker, T_*ACT1*_-*tHMG1*-P_*GAL10*_-P_*GAL1*_-Tm*CrtE*-T_*GPM1*_	This study
pCY09	pJET1.2/blunt possessing *LEU2* homologous arm with *LEU2* marker, T_*CYC1*_-*RFP*	This study
pCY10	pJET1.2/blunt possessing *LEU2* homologous arm with *LEU2* marker, T_*CYC1*_-*RFP*-P_*GAL3*_	This study
pCY11	pJET1.2/blunt possessing *LEU2* homologous arm with *LEU2* marker, T_*CYC1*_-*RFP*-P_*GAL7*_	This study
pCY12	pJET1.2/blunt possessing *LEU2* homologous arm with *LEU2* marker, T_*CYC1*_-*RFP*-P_*GAL10*_	This study
pCY13	pJET1.2/blunt possessing *LEU2* homologous arm with *LEU2* marker, T_*CYC1*_-Bt*CrtI*-P_*GAL3*_-T_*ACT1*_-*tHMG1*-P_*GAL10*_-P_*GAL1*_-Tm*CrtE*-T_*GPM1*_	This study
pCY14	pJET1.2/blunt possessing *LEU2* homologous arm with *LEU2* marker, T_*CYC1*_-Bt*CrtI*-P_*GAL7*_-T_*ACT1*_-*tHMG1*-P_*GAL10*_-P_*GAL1*_-Tm*CrtE*-T_*GPM1*_	This study
pCY15	pJET1.2/blunt possessing *YGLCtau3* homologous arm with *HphMX* marker, P_*GAL7*_-Bt*CrtI*-T_*CYC1*_	This study
pCY40	pJET1.2/blunt possessing *YPRCdelta15* homologous arm with *HphMX* marker, P_*GAL1*_-*INO2*-T_*CPS1*_	This study

### Assay of extracellular glucose, ethanol, acetate and glycerol

The concentrations of residual glucose, ethanol, acetate and glycerol in the medium were determined by HPLC (Waters Corp., USA) with a refractive index detector. Aminex HPX-87H column (BioRad, CA) was used for separation at the column temperature of 65 °C. 5 mM H_2_SO_4_ was used as eluent with a flow rate of 0.6 mL/min.

### Assay of acetyl-CoA

Cells were sampled during the course of lycopene shake-flask fermentation for acetyl-CoA assay. Acetyl-CoA was extracted as previously described [22] and analyzed by the acetyl-CoenzymeA Assay Kit (Sigma-Aldrich). The acetyl-CoA concentration was normalized by dry cell weight.

### Assay of promoter strength

Fluorescence intensity of red fluorescence protein (RFP) was used to characterize the strengths of *GAL3*, *GAL7* and *GAL10* promoters as previously described [23]. The strain without promoter fused with RFP (SyBE_Sc14C40) was used as the negative control. Culturing procedures of all the test strains (SyBE_Sc14C40–SyBE_Sc14C43; Table 1) were the same as lycopene fermentation in shake-flasks. Every 6 h of cultivation, cells were harvested, washed and diluted with phosphate-buffered saline (PBS) into an OD_600_ of 0.3–0.4 for fluorescence assay. RFP fluorescence intensity was detected by SpectraMax M2 microplate reader with excitation and emission wavelengths at 587 and 611 nm, respectively. Promoter strength was determined as the ratio of the fluorescence to OD_600_ for each strain.

### Extraction and analysis of carotenoid

Extraction of carotenoid was as described by Xie et al. [24] with some modifications. Briefly, cells harvested from cultures were washed, resuspended in boiling 3 N HCl for 2 min, and cooled in an ice-bath for 3 min. Then, cells debris were washed twice with water, resuspended in acetone containing 1 % BHT (w/v), vortexed with glass beads (425–600 µm, Sigma) until colorless, and followed by centrifugation. The acetone phase containing the extracted carotenoid was filtered for HPLC analysis. A HPLC system (Waters e2695) equipped with a BDS Hypersil C18 column (4.6 × 150 mm, 5 µm) and a UV/VIS detector (Waters 2489) was used to analyze the produced carotenoid. The signals of phytoene, phytofluene, ζ-carotene, neurosporene and lycopene were detected at 287, 349, 401, 440 and 471 nm, respectively [25]. The mobile phase consisted of methanol–acetonitrile-dichloromethane (21:21:8 v/v) with a flow rate of 1 mL/min at 30 °C [26]. Total carotenoid was calculated as the sum of the above carotenoids.

### Microscopy

Microscopic analysis was used to investigate lycopene formation of strain SyBE_Sc14C45 during shake-flask fermentation with YPDG medium. SyBE_Sc14C45 cultivated in YPD medium (without galactose) was used as control. After 36 h of cultivation, cells were harvested, washed and diluted with sterile water into an OD_600_ of 5.0. Images were taken with an Olympus CX41 (Olympus, Tokyo, Japan).

### Fed-batch fermentation

Strain SyBE_Sc14D14 was selected for fed-batch fermentation. Seed cultures were prepared by inoculating 250 µL of glycerol-stock into a 250 mL shake-flask containing 25 mL YPD and culturing at 30 °C for 16 h to an OD_600_ of 6–7, and then 15 mL of precultures were inoculated into a 2 L shake-flask containing 400 mL YPD and subcultured for an additional 8 h at 30 °C to an OD_600_ of 5–6. Seed cultures were transferred into a 5 L bioreactor (BaiLun, China) containing 2 L YPD batch medium at a 10 % (v/v) inoculum. Fermentation was carried out at 30 °C with an air flow rate of 1.5 vvm. The dissolved oxygen was kept at 30 % by adjusting the agitation speed from 400 to 700 rpm and pH was controlled at 6.0 by automatic addition of 6 M sodium hydroxide.

According to the employed galactose-inducible system for lycopene biosynthesis, fed-batch fermentation was divided into two stages: cell growth stage and lycopene production stage. During the first stage to achieve maximal cell growth, concentrated glucose solution (500 g/L) was fed periodically into bioreactors to keep the glucose concentration under 2 g/L. In the meanwhile, 100 mL of the concentrated yeast extract solution (400 g/L) was fed periodically into the bioreactor every 10 h. Once cell growth entered stationary phase, glucose and yeast extract feedings were ceased, and 10 g/L of D-(+)-galactose was added to induce lycopene biosynthesis. After the depletion of the residual glucose, cells began to consume ethanol converted by glucose consumption. Ethanol concentration was controlled below 5 g/L by adjusting 100 % ethanol feeding rate until harvest.

## Results and discussion

### Construction of inducible lycopene biosynthesis pathway

To avoid the potential toxicity of lycopene, genes responsible for carotenoid synthesis were placed under the control of galactose-regulated *GAL* promoters. Δ*gal1* Δ*gal7* Δ*gal10* and Δ*gal80* were two routine strategies to employ *GAL* promoters, since Δ*gal1* Δ*gal7* Δ*gal10* eliminates galactose utilization and Δ*gal80* does not require galactose for induction [27]. Here, *S. cerevisiae* SyBE_Sc14C01 (Δ*gal80*) and SyBE_Sc14C02 (Δ*gal1* Δ*gal7* Δ*gal10*) were chosen as the host cells for lycopene production. The carotenogenic pathway was constructed by genomic integration of *CrtE*, *CrtB*, *CrtI* and *tHMG1* in the respective hosts (Fig. 1a). As a result, strain SyBE_Sc14C07 (Δ*gal1* Δ*gal7* Δ*gal10*) with CrtE and CrtB from *Pantoea agglomerans* and CrtI from *Blakeslea trispora* produced 78.8 % higher lycopene yield (4.31 mg/g DCW) than strain SyBE_Sc14C06 (Δ*gal80*) harboring the same *Crt* genes (2.43 mg/g DCW) after 48 h of shake-flask culture in YPDG medium (Additional file 1: Figure S2). Thus, strain SyBE_Sc14C02 (Δ*gal1* Δ*gal7* Δ*gal10*) was selected as the host cell for the further study.

### Enhancement of acetyl-CoA pool by the deletion of *YPL062* *W*

**Fig. 2:**
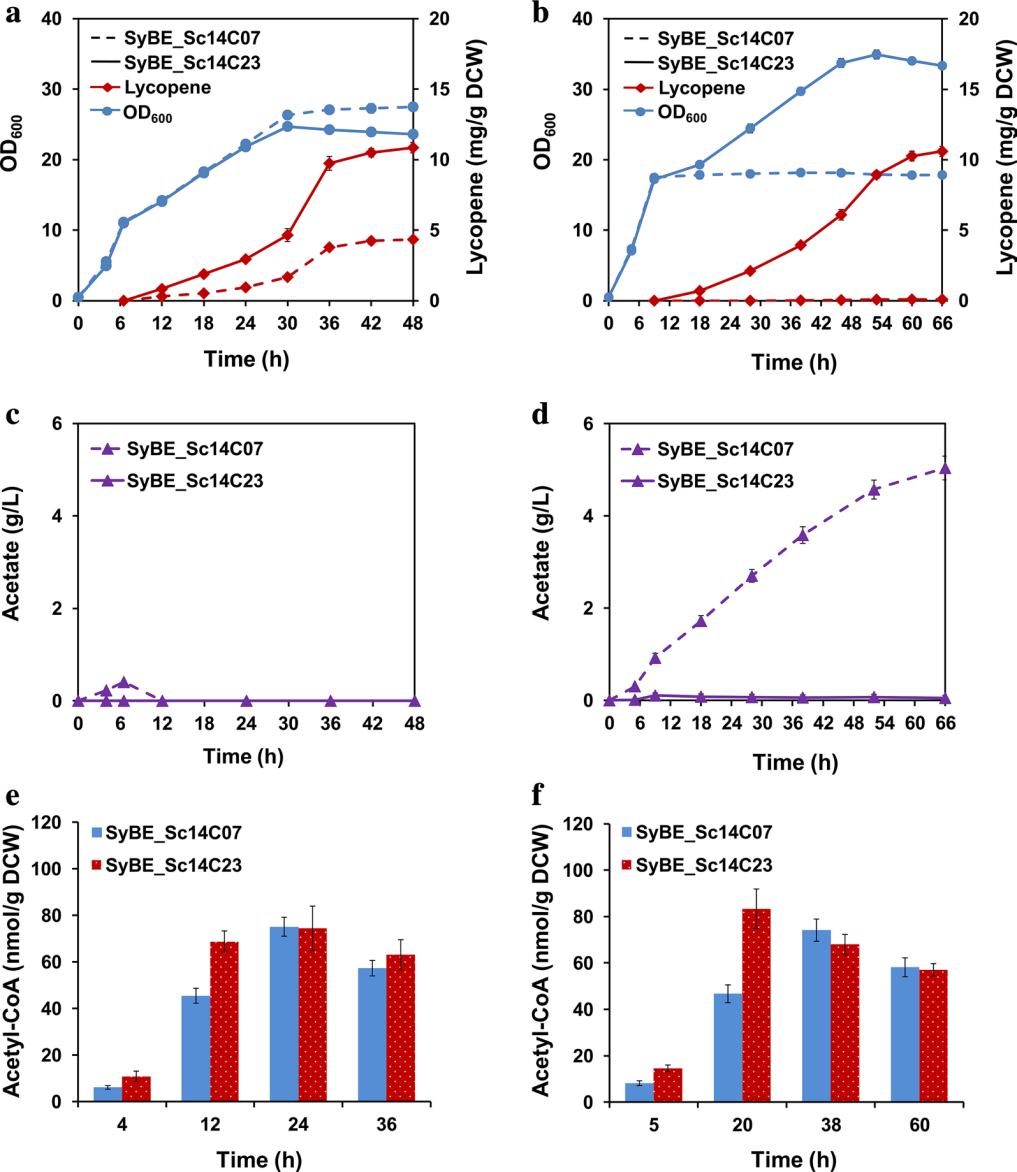
The effect of Δ*ypl062w* on lycopene production. *S. cerevisiae* SyBE_Sc14C07 and SyBE_Sc14C23 were cultivated in YPDG media containing different concentrations of glucose (2 %, *left side*; 4 %, *right side*), respectively, in shake-flasks for analysis of lycopene production (**a**, **b**), acetate accumulation (**c**, **d**) and cytosolic acetyl-CoA level (**e**, **f**). The *error bars* represent standard deviation calculated from triplicate experiments

Δ*ypl062w* (Additional file 1: Table S2) was previously reported to enhance carotenoid production by increasing the intracellular mevalonate level [10], but the mechanism was not clear. In order to testify whether Δ*ypl062w* is benefit to lycopene production, *YPL062W* was deleted in SyBE_Sc14C07, generating strain SyBE_Sc14C23. As a result, Δ*ypl062w* increased lycopene yield by more than 1.5-fold when cultured in YPDG medium with 2 % glucose (Fig. 2a), which is consistent with previous work [10]. Compared to strain SyBE_Sc14C07 with 0.51 g/L extracellular acetate accumulation, no acetate accumulation was observed in strain SyBE_Sc14C23 (Fig. 2c). Furthermore, SyBE_Sc14C07 and SyBE_Sc14C23 were cultivated in YPDG media with higher (4 %) glucose. As shown in Fig. 2b and d, 10.84 mg/g DCW of lycopene together with very little amount of acetate was detected in SyBE_Sc14C23, whereas only 70 µg/g DCW of lycopene and up to 5.04 g/L of acetate were obtained in SyBE_Sc14C07. It was also observed that cell growth of strain without Δ*ypl062w* was abolished when acetate accumulated up to 1.0 g/L (Fig. 2d), which is in accordance with Thomas et al. [28]. Moreover, when SyBE_Sc14C07 was cultured in YPDG medium with 2 % glucose, different concentrations (0, 0.5, 1.0, 1.5 g/L) of acetic acid were added to the media at the time of 7 h when glucose was exhausted (indicated as arrow in Additional file 1: Figure S3). Cell growth was also abolished when the added acetic acid concentration exceeded 1.0 g/L (Additional file 1: Figure S3A). Lycopene yield was dropped by 98.4 % (from 4.31 mg/g DCW to 67 µg/g DCW) when additional 0.5 g/L acetic acid was added (Additional file 1: Figure S3B). No lycopene production was detected when 1.0 g/L acetic acid was added (Additional file 1: Figure S3B), suggesting acetate accumulation would be harmful to lycopene biosynthesis. Thus, Δ*ypl062w* acted as an important role in *S. cerevisiae* to reduce acetate accumulation.

As is known, acetate is the direct precursor for cytosolic acetyl-CoA. Therefore, we assumed that Δ*ypl062w* would enhance cytosolic acetyl-CoA pool from acetate. As expected, the cytosolic acetyl-CoA concentrations in SyBE_Sc14C23 were increased by approximately 100 % than those in SyBE_Sc14C07 at early times (Fig. 2e, f). When lycopene was rapidly accumulated, SyBE_Sc14C23 demonstrated the same cytosolic acetyl-CoA level but higher lycopene production as compared to SyBE_Sc14C07 (Fig. 2a, b, e, f). This result suggested the expansion on lycopene production might be derived from the increase in acetyl-CoA supplement, since the intracellular mevalonate level of Δ*ypl062w* strain was significantly increased during terpenoid production [10]. Therefore, Δ*ypl062w* enhanced “trapping” the carbon from acetate accumulation toward acetyl-CoA, which consequently improved MVA pathway flux and further lycopene production. Our findings made us a better understanding of the effect of Δ*ypl062w* on MVA pathway.

### Optimal combination of CrtE, CrtB and CrtI by screening enzymes from diverse sources

**Fig. 3:**
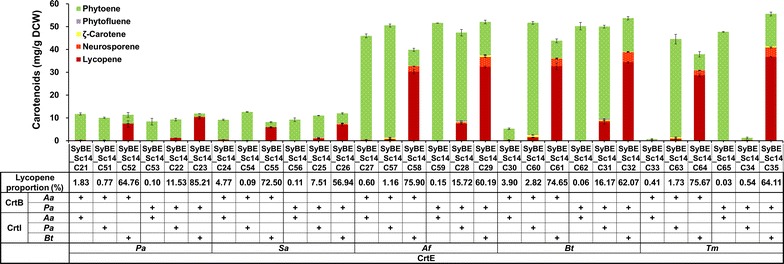
Combinatorial optimization of CrtE, CrtB and CrtI from diverse species. Thirty lycopene-producing strains were constructed by screening enzymes from various sources and tested for lycopene production. Pa, *Pantoea agglomerans*; Sa, *Sulfolobus acidocaldarius*; Af, *Archaeoglobus fulgidus*; Bt, *Blakeslea trispora*; Tm, *Taxus x media*; Aa, *Paracoccus* sp. (formerly *Agrobacterium aurantiacum*). The *error bars* represent standard deviation calculated from triplicate experiments

It is known that enzymes from different organisms often vary in catalytic activities [29, 30]. Besides, host cell compatibility may also affect the optimal performance of heterologous enzymes [16, 31]. Thus, screening enzymes from diverse sources offers an effective strategy to increase the productivity of heterologous pathway in specific host [29, 32]. To date, most of the carotenogenic genes employed in heterologous biosynthesis were derived from *Panto*ea, *Paracoccus* or *Xanthophyllomyces* species [33, 34]. However, in *S. cerevisiae*, the currently reported *Crt* genes for high-level carotenoid production were only from *X. dendrorhous* [16, 24, 35]. In this study, we aimed to rebuild a carotenogenic pathway with high productivity in *S. cerevisiae* by screening enzymes (CrtE, CrtB, and CrtI) from some other species except *X. dendrorhous*. Five CrtEs originated from *P. agglomerans* (PaCrtE), *Sulfolobus acidocaldarius* (SaCrtE), *Archaeoglobus fulgidus* (AfCrtE), *B. trispora* (BtCrtE) and *Taxus x media* (TmCrtE), two CrtBs from *P. agglomerans* (PaCrtB) and *Paracoccus* sp. (formerly *Agrobacterium aurantiacum*) (AaCrtB), and three CrtIs from *P. agglomerans* (PaCrtI), *Paracoccus* sp. (AaCrtI) and *B. trispora* (BtCrtI) were selected for carotenoid biosynthesis. As illustrated in Fig. 3, thirty lycopene-producing strains were constructed and their production was investigated. Consequently, the lycopene yield in strain SyBE_Sc14C35 harboring the best enzyme combination (TmCrtE, PaCrtB and BtCrtI) was increased by 7.5-fold, up to 36.75 mg/g DCW, and the lycopene proportion in carotenoid was 64.11 % (Fig. 3). This strain was used as the candidate for the further optimization.

In general, CrtE, one of the rate-limiting enzymes in carotenoid pathway [36], was found to be crucial to the production yield of overall carotenoid. As shown in Fig. 3, strains harboring Af*CrtE*, Bt*CrtE* or Tm*CrtE* showed much higher yield of total carotenoid than that harboring Pa*CrtE* or Sa*CrtE.* Tm*CrtE* was reported to possess a larger pocket and higher affinity for farnesyl diphosphate (FPP) binding than other GGPPSs [30], thus it was reasonable that strain with Tm*CrtE* achieved high carotenoid yield. *CrtE* from *B. trispora* [37] was firstly well expressed in *S. cerevisiae* and achieved quite high yield of overall carotenoid, demonstrating that BtCrtE would be a promising GGPPS candidate for further study. AfCrtE can directly utilize dimethylallyl pyrophosphate (DMAPP)/isopentenyl pyrophosphate (IPP) to synthesize geranylgeranyl diphosphate (GGPP) and thus avoid competing FPP for sterols biosynthesis [38], which might explain the improved carotenoid production catalyzed by AfCrtE. In addition to AfCrtE, SaCrtE was also a bifunctional FPP/GGPP synthase [39] and has been demonstrated to increase diterpenoids production [40, 41]. But it was difficult to interpret the low carotenoid yield by SaCrtE according to our current data, which might be ascribed to the insufficient expression of Sa*CrtE* or its incompatibility with *CrtB* and *CrtI*.

Phytoene, synthesized by CrtB from GGPP, is the first intermediate of the carotenoid pathway. Then lycopene was generated by CrtI through four successive dehydrogenation steps from phytoene (Fig. 1a). As illustrated in Fig. 3, rather than lycopene, phytoene was one of the major components of the total carotenoid in most of our engineered strains, indicating that CrtI-catalyzed conversion from phytoene to lycopene was another rate-limiting step, which is consistent with previous reports [16, 35]. To be noted, all the strains harboring Bt*CrtI* showed much better performance than strains harboring Pa*CrtI* or Aa*CrtI*, irrespective of lycopene yield or proportion in carotenoid (Fig. 3). BtCrtI, an enzyme from eukaryotic organsims, was firstly well expressed in *S. cerevisiae* and found to be more suitable for high-level conversion from phytoene to lycopene in *S. cerevisise* according to our results. The molecular mechanism for its high efficiency in conversion from phytoene to lycopene is an interesting topic in the field in future.

### Fine-tuning of Bt*CrtI* and selection of homologous haploid yeast hosts

**Fig. 4:**
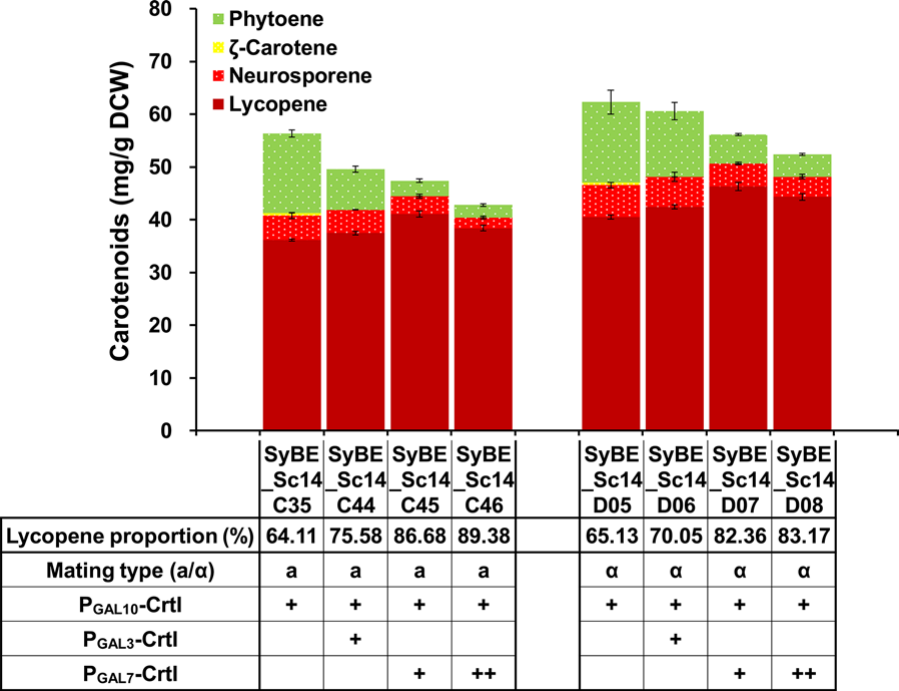
Carotenoid production by fine-tuning of Bt*CrtI* and selecting homologous haploid strains. Bt*CrtI* expression was fine-tuned by adjusting copy number and promoter strength, and haploid cells with different mating types (*a*, *α*) were compared as well for carotenoid production. The *error bars* represent standard deviation calculated from triplicate experiments

Despite the improved lycopene yield in SyBE_Sc14C35, approximately 26 % of the total carotenoid was phytoene, which indicated that the phytoene conversion directed by Bt*CrtI* was not efficient enough. In order to achieve higher lycopene proportion, the expression level of Bt*CrtI* needs to be fine-tuned. Here, Bt*CrtI* was fine-tuned by adjusting different promoters and integration copy numbers. The strengths of *GAL* promoters used in this step were characterized in strain SyBE_Sc14C10 (CEN.PK2-1C, Δ*gal1* Δ*gal7* Δ*gal10*, Δ*ypl062w*), and P_*GAL3*_ was found to be the weakest one (Additional file 1: Figure S4). When one additional copy of Bt*CrtI* under the control of P_*GAL3*_ or P_*GAL7*_ was integrated in strain SyBE_Sc14C35, the lycopene proportion in the resulting strain SyBE_Sc14C44 or SyBE_Sc14C45 was increased by 17.9 % (from 64.11 to 75.58 %) or 35.2 % (up to 86.68 %), respectively (Fig. 4). Moreover, a slight decrease in lycopene yield was observed after another additional copy of P_*GAL7*_-Bt*CrtI* integrated in SyBE_Sc14C45 (Fig. 4), which is similar to recent report [16]. To be noted, although the lycopene yield was modestly increased, the total carotenoid yield was decreased obviously after fine-tuning steps for higher lycopene proportion (Fig. 4). As shown in Additional file 1: Figure S5, most of the lycopene was accumulated in cell membrane, which is consistent with early reports [42, 43]. Rapid lycopene accumulation in cell membrane would lead to membrane stress or cell toxicity, which might explain the significant decrement in the content of total carotenoid after fine-tuning. Therefore, increasing lycopene tolerance in *S. cerevisiae* would be an effective direction to improve lycopene yield.

Haploid cell type was reported to have significant influence on heterologous terpenoid production [44]. Jackson et al. [45] found that *MATa* strain was more suitable to produce epi-cedrol than *MATα* strain, since *MATa* strain might synthesize more FPP for prenylation of the mating pheromone [46]. However, cell mating types did not have significant effect on linalool production [47]. Here, lycopene production in CEN.PK2-1C (*MATa*) and CEN.PK2-1D (*MATα*) with the same genetic modifications was evaluated, respectively. The strains (SyBE_Sc14D05–SyBE_Sc14D08) derived from CEN.PK2-1D (*MATα*) achieved 12–15 % higher lycopene yield than strains (SyBE_Sc14C35, SyBE_Sc14C44–SyBE_Sc14C46) from CEN.PK2-1C (*MATa*) (Fig. 4), suggesting that *MATα* strain was preferred in the case of lycopene production. Finally, the strain SyBE_Sc14D07 (*MATα*) achieved lycopene yield of 46.26 mg/g DCW with a proportion of 82.36 % (Fig. 4).

### Effects of distant genetic loci on lycopene production

**Fig. 5:**
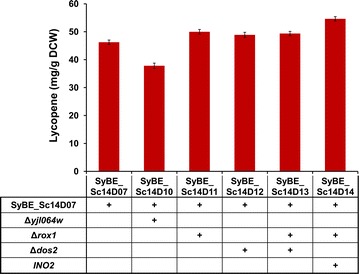
The effects of distant genetic loci on lycopene production. Three gene-deletion targets (*ROX1*, *DOS2*, and *YJL064W*) and one overexpression target (*INO2*) were investigated in *S. cerevisiae* SyBE_Sc14D07 for lycopene production. The *error bars* represent standard deviation calculated from triplicate experiments

As the engineered metabolic pathways were highly inter-connected with the rest of cellular metabolism and tightly regulated [48], distantly located genetic loci in host cell could also have potential interactions with target pathway. As Δ*rox1*, Δ*dos2* and Δ*yjl064w* (Additional file 1: Table S2) were proved to greatly benefit carotenoid production [5, 10], these three distant genetic loci were knocked out individually in strain SyBE_Sc14D07. As shown in Fig. 5, both Δ*rox1* and Δ*dos2* conferred a modest increase (8.7 and 5.7 %, respectively) in lycopene production as expected, while Δ*yjl064w* led to 18.2 % decreased lycopene yield as compared to SyBE_Sc14D07. Moreover, the combination of Δ*rox1* and Δ*dos2* did not show a synergistic effect on lycopene production (Fig. 5), which is inconsistent with the results obtained by Trikka et al. [5]. These inconsistencies might be attributed to that the impact of perturbations in one strain may not be directly applied to another strain with a modified genetic background [1]. Consequently, a relatively higher lycopene yield of 50.28 mg/g DCW was obtained in SyBE_Sc14D11 with Δ*rox1*.

As shown in Additional file 1: Figure S5, lycopene tended to accumulate in cell membrane and thus cause cell toxicity. Here, *INO2* (Additional file 1: Table S2), an endogenous transcription factor related to cellular stress response [49, 50], was overexpressed in SyBE_Sc14D11. As a result, a lycopene yield of 54.63 mg/g DCW, the highest yield reported, was obtained in the resulting strain SyBE_Sc14D14 in shake-flask cultivation (Fig. 5). INO2 was reported to alleviate alkanes (C9–C11) toxicity by regulating genes associated with efflux pumps, stress response, lipid metabolism and ergosterol biosynthesis in *S. cerevisiae* [51]. High-level expression of *INO2* was proved to up-regulate phospholipid and sterol biosynthesis [52]. Therefore, increasing lycopene tolerance through modifying membrane components (i.e. lipid, and ergosterol) may be the main reason for the improvement of lycopene yield via INO2. Therefore, *INO2* was identified as a novel target for lycopene production in *S. cerevisiae*. Strain SyBE_Sc14D14 was chosen for fed-batch fermentation.

### Lycopene overproduction in fed-batch fermentation

**Fig. 6:**
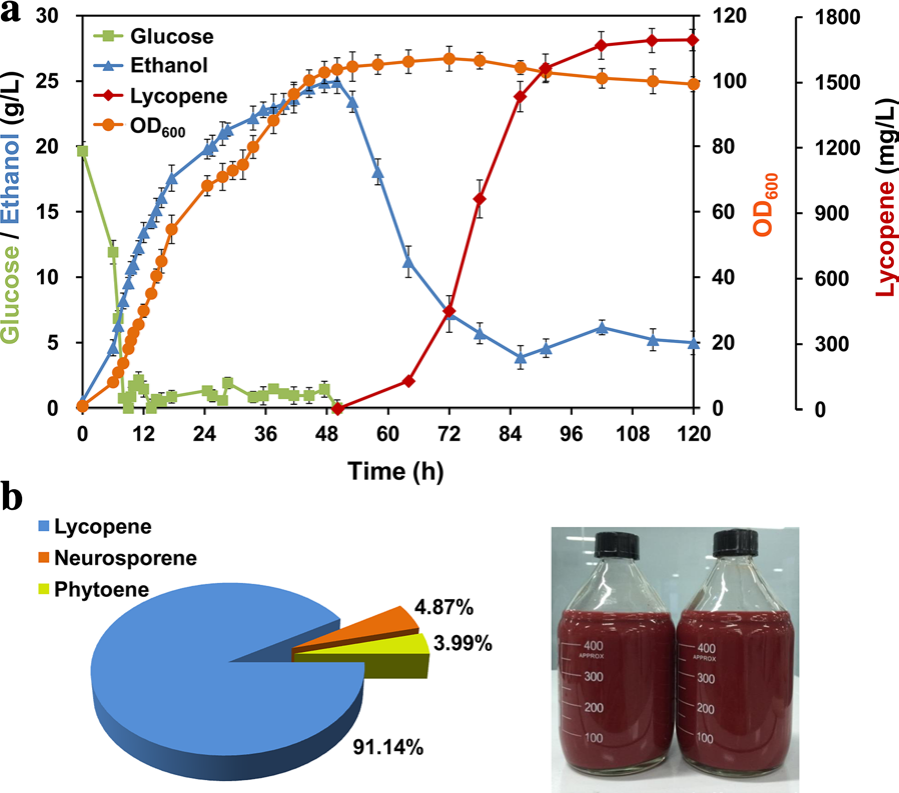
Lycopene production in fed-batch fermentation. **a** Profile of glucose, ethanol, cell density and lycopene accumulation of strain SyBE_Sc14D14 during fed-batch fermentation. **b** Percentage ratios of the produced carotenoid composition at 120 h. The *red liquid* in the bottles was the lycopene fermentation broth. The *error bars* represent standard deviation calculated from duplicate experiments

To evaluate the production performance of the engineered strain SyBE_Sc14D14, fed-batch fermentation was performed at a 2 L scale using YPD as the medium (Fig. 6). Based on carbon restriction strategy, trace amount of acetate was observed during the whole process (Additional file 1: Figure S6). Eventually, a total carotenoid titer of 1.81 g/L (60.94 mg/g DCW), consisting of 3.99 % of phytoene, 4.87 % of neurosporene and 91.14 % of lycopene, was obtained after 120 h of cultivation (Fig. 6). Lycopene yield of 55.56 mg/g DCW achieved in our work was the highest yield in yeast strains to date. However, the lycopene titer (1.65 g/L) was just similar to previous work by Xie et al. [16]. This is due to the relative low cell density, since the highest OD_600_ only reached 106 throughout the fermentation. Additionally, excessive accumulations of ethanol and glycerol were also observed during cell growth phase (Fig. 6a; Additional file 1: Figure S6), which competed carbon flow from biomass synthesis and implied redox imbalance of our engineered strain [53]. In future, to limit glucose below 0.5 g/L in growth phase will greatly increase cell density as well as reduce by-product (ethanol or glycerol) accumulation. Moreover, intracellular and extracellular metabolomics analysis will be an efficient way to find some biomarkers for batch media or feeding solution optimization. Off-gas analysis will also be promising for more precise process control. As recent studies in media optimization have demonstrated great potential in lycopene overproduction [16, 54], we believe that lycopene production by our engineered strain would be further improved by continuous efforts in both metabolic engineering and fermentation optimization.

## Conclusions

In this work, lycopene overproduction was realized by combinatorial engineering of *S. cerevisiae* and lycopene biosynthesis pathway. Extracellular acetate accumulation was reduced and cytosolic acetyl-CoA pool was enhanced through the deletion of *YPL062W*. A novel and optimal combination of CrtE, CrtB and CrtI was obtained by screening enzymes from diverse sources. It was also found that CrtI from *B. trispora* had significant influence on lycopene yield as well as proportion in carotenoid. The proportion of lycopene was significantly increased via fine-tuning of *CrtI*. Then the effects of cell mating types, several potential distant targets (*YJL064* *W*, *ROX1*, and *DOS2*), and *INO2*, a stress-related transcription factor, were also investigated. Lycopene yield was stepwise improved by approximately 22-fold as compared to the starting strain. The highest reported lycopene yield (55.56 mg/g DCW) and titer (1.65 g/L) were achieved in 5-L bioreactors, providing a good example for microbial overproduction of pharmaceutical and chemical products through combinatorial engineering of host cell and heterologous pathway.

## Additional file

10.1186/s12934-016-0509-4 This file consists of two supplemental tables and seven supplemental figures. **Table S1**. Oligonucleotides used in this study. **Table S2**. Distant genetic loci involved in this study. **Figure S1**. Integration modules constructed in this study. **Figure S2**. Comparison of Δ*gal1 *Δ*gal7* Δ*gal10* strain and Δ*gal80* strain on lycopene production. **Figure S3**. The effects of acetic acid addition on cell growth (A) and lycopene production (B) in strain SyBE_Sc14C07. **Figure S4**. Time course of promoter strengths. **Figure S5**. Microscopic images of lycopene-producing strain. **Figure S6**. Profile of glycerol and acetate accumulation. **Figure S7**. Sequences of codon-optimized genes.

## Abbreviations

MVA: mevalonate
HMG-CoA: 3-hydroxy-3-methylglutaryl coenzyme A
IPP: isopentenyl pyrophosphate
DMAPP: dimethylallyl pyrophosphate
FPP: farnesyl diphosphate
GGPP: geranylgeranyl diphosphate
tHMG1: truncated 3-hydroxy-3-methylglutaryl coenzyme A reductase
CrtE: geranylgeranyl diphosphate synthase (GGPPS)
CrtB: phytoene synthase
CrtI: phytoene desaturase
Aa: *Paracoccus* sp. (formerly *Agrobacterium aurantiacum*)
Pa: *Pantoea agglomerans*
Sa: *Sulfolobus acidocaldarius*
Af: *Archaeoglobus fulgidus*
Bt: *Blakeslea trispora*
Tm: *Taxus x media*
