# Supplementary material for: Lycopene overproduction in Saccharomyces cerevisiae through combining pathway engineering with host engineering
Source: Microb Cell Fact. 2016 Jun 21;15:113. doi: 10.1186/s12934-016-0509-4 (PMC4915043; doi:10.1186/s12934-016-0509-4)
Supplement: Supplementary file 1 — 10.1186/s12934-016-0509-4 This file consists of two supplemental tables and seven supplemental figures. Table S1. Oligonucleotides used in this study. Table S2. Distant genetic loci involved in this study. Figure S1. Integration modules constructed in this study. Figure S2. Comparison of Δgal1 Δgal7 Δgal10 strain and Δgal80 strain on lycopene production. Figure S3. The effects of acetic acid addition on cell growth (A) and lycopene production (B) in strain SyBE_Sc14C07. Figure S4. Time course of promoter strengths. Figure S5. Microscopic images of lycopene-producing strain. Figure S6. Profile of glycerol and acetate accumulation. Figure S7. Sequences of codon-optimized genes. [file 12934_2016_509_MOESM1_ESM.docx]

**Supporting information**

**Lycopene overproduction in *Saccharomyces cerevisiae* through combining pathway engineering with host engineering**

Yan Chen^a,b^, Wenhai Xiao^a,b^*, Yin Wang^a,b^, Hong Liu^a,b^, Xia Li^a,b^, Yingjin Yuan^a,b^

^a^ Key Laboratory of Systems Bioengineering (Ministry of Education), Tianjin University, Tianjin, 300072, PR China.

^b^ SynBio Research Platform, Collaborative Innovation Center of Chemical Science and Engineering (Tianjin), School of Chemical Engineering and Technology, Tianjin University, Tianjin, 300072, PR China.

*Corresponding author: Wenhai Xiao (Email: wenhai.xiao@tju.edu.cn, Tel: 86-22-60973987，Postal address: No. 92, Weijin Road, Nankai District, Tianjin, 300072, PR China)

**Table S1.** Oligonucleotides used in this study

| **Oligo name** | **Sequence (5’- 3’)** | |
| --- | --- | --- |
| **For construction of T*_CYC1_*-*CrtI*-P*_GAL10_*-P*_GAL1_*-*CrtB*-T*_PGK1_*, *TRP1* homologous arm** | | |
| TPR1_LF | *GTTTAAAC*GGAAGAGGAGTAGGGAA | |
| TPR1_LR | TACGATGCTGTTCTATTAAATGCT | |
| CYC1t_F | GGCCGCATCATGTAATTAG | |
| CYC1t_R | AGCATTTAATAGAACAGCATCGTAGCAAATTAAAGCCTTCGAG | |
| *Bt*CrtI_F | GTAAGAATTTTTGAAAATTCAATATAAATGTCTGATCAGAAGAAGCACA | |
| *Bt*CrtI_R | CTAATTACATGATGCGGCCTTATATCCTAATATCGTTAGAGTTCTG | |
| *Pa*CrtI_F | GTAAGAATTTTTGAAAATTCAATATAAATGAAGAAGACAGTCGTCATAGG | |
| *Pa*CrtI_R | CTAATTACATGATGCGGCCTTACTGCAAGTCCTCTATCATCA | |
| *Aa*CrtI_F | GTAAGAATTTTTGAAAATTCAATATAAATGAACGCTCACTCCCCT | |
| *Aa*CrtI_R | CTAATTACATGATGCGGCCTTATGCGGAAGCCAAATC | |
| GAL10p_F | TTATATTGAATTTTCAAAAATTCTTAC | |
| GAL1p_R | TATAGTTTTTTCTCCTTGACGTTA | |
| *Pa*CrtB_F | TAACGTCAAGGAGAAAAAACTATAATGTCACAACCACCATTATTG | |
| *Pa*CrtB _R | CTATCGATTTCAATTCAATTCAATTTAAACAGGTCTTTGCCATAA | |
| *Aa*CrtB_F | TAACGTCAAGGAGAAAAAACTATAATGTCCGACTTAGTTTTGACC | |
| *Aa*CrtB_R | CTATCGATTTCAATTCAATTCAATTTAGGCGTGATGTGGTCTT | |
| PGK1t_F | ATTGAATTGAATTGAAATCGATAG | |
| PGK1t_R | CGTCATAACTGCAAAGTACACATATATAACGAACGCAGAATTTTCG | |
| TPR1_RF | ATATATGTGTACTTTGCAGTTATGACG | |
| TPR1_RR | *GTTTAAAC*ACGCCAACCAAGTATTT | |
| **PCR verification of T*_CYC1_*-*CrtI*-P*_GAL10_*-P*_GAL1_*-*CrtB*-T*_PGK1_*, *TRP1* homologous arm** | | |
| TPR1_VF | AGACATGGAGGGCGTTATTA | |
| GAL1p_VR | CTTTATTGTTCGGAGCAGTG | |
| GAL10p_VF | CGCTTAACTGCTCATTGCTAT | |
| TPR1_VR | AGTTTGATTCCATTGCGGT | |
| **For construction of T*_ACT1_-tHMG1-*P*_GAL10_-*P*_GAL1_-CrtE-*T*_GPM1_*, *LEU2* homologous arm with *LEU2* marker** | | |
| LEU2_LF | *GTTTAAAC*ATAACGAGAACACACAGGG | |
| LEU2_LR | ATCATTAAAGTAACTTAAGGAGTTAAATTTAAGCAAGGATTTTCTTAACTTC | |
| TDH2t_F | ATTTAACTCCTTAAGTTACTTTAATGAT | |
| TDH2t_R | GCGAAAAGCCAATTAGTGT | |
| ACT1t_F | TCTCTGCTTTTGTGCGC | |
| ACT1t_R | ACACTAATTGGCTTTTCGCTACACGGTCCAATGGATAAAC | |
| tHMG1_F | GTAAGAATTTTTGAAAATTCAATATAAATGGTTTTAACCAATAAAACAGTC | |
| tHMG1_R | GCGCACAAAAGCAGAGATTAGGATTTAATGCAGGTGAC | |
| GAL10p_F | TTATATTGAATTTTCAAAAATTCTTAC | |
| GAL1p_R | TATAGTTTTTTCTCCTTGACGTTA | |
| *Pa*CrtE_F | TAACGTCAAGGAGAAAAAACTATAATGGTTTCTGGTTCTAAGGCT | |
| *Pa*CrtE_R | CATCAAATCATTCATTCTTCAGACTTAAGCTATCTTCATGACAGGTG | |
| *Sa*CrtE_F | TAACGTCAAGGAGAAAAAACTATAATGTCTTACTTTGATAATTACTTTAATGA | |
| *Sa*CrtE_R | CATCAAATCATTCATTCTTCAGACTTACTTCCTTCTTCTAATTGTAAATTC | |
| *Af*CrtE_F | TAACGTCAAGGAGAAAAAACTATAATGTTGAAAGAAGAAATTGCTAAA | |
| *Af*CrtE_R | CATCAAATCATTCATTCTTCAGACTTATTTCTTTCTAGTAACTAAAAAATCTGT | |
| *Bt*CrtE_F | TAACGTCAAGGAGAAAAAACTATAATGTTGACTTCTTCTAAATCTATTGA | |
| *Bt*CrtE_R | CATCAAATCATTCATTCTTCAGACTTATTCGTTGACAGACAAGACG | |
| *Tm*CrtE_F | TAACGTCAAGGAGAAAAAACTATAATGGCTTATACCGCAATGG | |
| *Tm*CrtE_R | CATCAAATCATTCATTCTTCAGACTTAGTTTTGCCTGAAAGCG | |
| GPM1t_F | GTCTGAAGAATGAATGATTTGATG | |
| GPM1t_R | CAAATATCATAAAAAAAGAGAATCTTTTATTCGAACTGCCCATTCA | |
| LEU2_RF | AAAGATTCTCTTTTTTTATGATATTTG | |
| LEU2_RR | *GTTTAAAC*TCCATCAAATGGTCAGG | |
| **PCR verification of T*_ACT1_-tHMG1-*P*_GAL10_-*P*_GAL1_-CrtE-*T*_GPM1_*, *LEU2* homologous arm with *LEU2* marker** | | |
| LEU2_VF | GGAATACTCAGGTATCGTAAGATGC | |
| GAL1p_VR | CTTTATTGTTCGGAGCAGTG | |
| GAL10p_VF | CGCTTAACTGCTCATTGCTAT | |
| LEU2_VR | CGTTAAGGCCGTTTCTGACA | |
| **For construction of P*_GAL3/7/10_ -RFP-*T*_CYC1_*, *LEU2* homologous arm with *LEU2* marker** | | |
| LEU2_LF | *GTTTAAAC*ATAACGAGAACACACAGGG | |
| TDH2t_R | GCGAAAAGCCAATTAGTGT | |
| CYC1t_RFP_F | CTGGTGGTATGGATGAATTATATAAATAAGGCCGCATCATGTAATTAGTT | |
| CYC1t_LEU2_R | ACACTAATTGGCTTTTCGCGCAAATTAAAGCCTTCGAGC | |
| RFP_F | ATGGTTTCAAAAGGTGAAGAAGAT | |
| RFP_R | TTATTTATATAATTCATCCATACCACCAG | |
| GAL3p_F | CAAATATCATAAAAAAAGAGAATCTTTTTGCTAGCCTTTTCTCGGTC | |
| GAL3p_R | ATCTTCTTCACCTTTTGAAACCATACTATGTGTTGCCCTACCTTTTTAC | |
| GAL7p_F | CAAATATCATAAAAAAAGAGAATCTTTGACGGTAGCAACAAGAATATAGCA | |
| GAL7p_R | ATCTTCTTCACCTTTTGAAACCATTTTTGAGGGAATATTCAACTGTTT | |
| GAL10p_F | CAAATATCATAAAAAAAGAGAATCTTTAGTGGTTATGCAGCTTTTCCA | |
| GAL10p_R | ATCTTCTTCACCTTTTGAAACCATTTATATTGAATTTTCAAAAATTCTTACTT | |
| LEU2_RFP_F | ATGGTTTCAAAAGGTGAAGAAGATAAAGATTCTCTTTTTTTATGATATTTG | |
| LEU2_RF | AAAGATTCTCTTTTTTTATGATATTTG | |
| LEU2_RR | *GTTTAAAC*TCCATCAAATGGTCAGG | |
| **PCR verification of P*_GAL3/7/10_ -RFP-*T*_CYC1_*, *LEU2* homologous arm with *LEU2* marker** | | |
| LEU2_VF | GGAATACTCAGGTATCGTAAGATGC | |
| LEU2_VR | CGTTAAGGCCGTTTCTGACA | |
| **For construction of T*_CYC1_-*Bt*CrtI-*P*_GAL3/7_-*T*_ACT1_-tHMG1-*P*_GAL10_-*P*_GAL1_-*Tm*CrtE-*T*_GPM1_*, *LEU2* homologous arm with *LEU2* marker** | | |
| LEU2_LF | *GTTTAAAC*ATAACGAGAACACACAGGG | |
| TDH2t_R | GCGAAAAGCCAATTAGTGT | |
| CYC1t_CrtI_F | CAGAACTCTAACGATATTAGGATATAAGGCCGCATCATGTAATTAG | |
| CYC1t_LEU2_R | ACACTAATTGGCTTTTCGCGCAAATTAAAGCCTTCGAGC | |
| *Bt*CrtI_LEU2_F | ATGTCTGATCAGAAGAAGCACA | |
| *Bt*CrtI_LEU2_R | TTATATCCTAATATCGTTAGAGTTCTG | |
| GAL3p_CrtI_F | GTTTATCCATTGGACCGTGTATTGCTAGCCTTTTCTCGGTC | |
| GAL3p_CrtI_R | TGTGCTTCTTCTGATCAGACATACTATGTGTTGCCCTACCTTTTTAC | |
| GAL7p_CrtI_F | GTTTATCCATTGGACCGTGTAGACGGTAGCAACAAGAATATAGCA | |
| GAL7p_CrtI_R | TGTGCTTCTTCTGATCAGACATTTTTGAGGGAATATTCAACTGTTT | |
| ACT1t_CrtI_R | TACACGGTCCAATGGATAAAC | |
| LEU2_RR | *GTTTAAAC*TCCATCAAATGGTCAGG | |
| **PCR verification of T*_CYC1_-*Bt*CrtI-*P*_GAL3/7_-*T*_ACT1_-tHMG1-*P*_GAL10_-*P*_GAL1_-*Tm*CrtE-*T*_GPM1_*, *LEU2* homologous arm with *LEU2* marker** | | |
| LEU2_VF | GGAATACTCAGGTATCGTAAGATGC | |
| CrtI_VR | GGTGCTCTTTCATTCACCAC | |
| GAL10p_VF | CGCTTAACTGCTCATTGCTAT | |
| LEU2_VR | CGTTAAGGCCGTTTCTGACA | |
| **For construction of P*_GAL7_-*Bt*CrtI-*T*_CYC1_*, *YGLCtau3* homologous arm with *HphMX* marker** | | |
| YGLC*tau3*_LF | *GTTTAAAC*GTTGTAGCAATTGTTGACG | |
| YGLC*tau3*_LR | ACAAACAGGGCAAATCAAGCA | |
| HphMX_F | TGCTTGATTTGCCCTGTTTGTTCTGTTTAGCTTGCCTCGTCC | |
| HphMX_R | GTTTTCGACACTGGATGGCG | |
| GAL7p_YGLC_F | CGCCATCCAGTGTCGAAAACGACGGTAGCAACAAGAATATAGCA | |
| CYC1t_YGLC_R | AAAAGCGAAGCTAACTAAGACAGCGCAAATTAAAGCCTTCGAG | |
| YGLC*tau3*_RF | GCTGTCTTAGTTAGCTTCGCTTTT | |
| YGLC*tau3*_RR | *GTTTAAAC*AGTACGATTTGCAGTTATGTAAA | |
| **PCR verification of P*_GAL7_-*Bt*CrtI-*T*_CYC1_*, *YGLCtau3* homologous arm with *HphMX* marker** | | |
| YGLC*tau3*_VF | | GGTAGTTCTCTTTCAACTGCTATTG |
| CYC1t_VR | | GCAAATTAAAGCCTTCGAG |
| CrtI_VR | | GGTGCTCTTTCATTCACCAC |
| YGLC*tau3*_VR | | AGCGTTCGTTCTATGCCTC |
| **For construction of P*_GAL1_-INO2-*T*_CPS1_*, *YPRCdelta15* homologous arm with *HphMX* marker** | | |
| YPRC*delta*15_LF | | *GTTTAAAC*TGTCCTGCAAATCGTGTA |
| YPRC*delta*15_LR | | TTTGCGAAACCCTATGCTCT |
| HphMX_YPRC_F | | AGAGCATAGGGTTTCGCAAATCTGTTTAGCTTGCCTCGTCC |
| HphMX_YPRC_R | | GGCGGCTTCTAATCCGTACTGTTTTCGACACTGGATGGCG |
| GAL1p_F | | AGTACGGATTAGAAGCCGCC |
| GAL1p_INO2_R | | TAATTCGTTCCCAGTTGCTTGGGACATTATAGTTTTTTCTCCTTGACGTTAAAG |
| INO2_F | | ATGTCCCAAGCAACTGGGAACGAATTA |
| INO2_R | | TCAGGAATCATCCAGTATGTGCT |
| CPS1t_F | | AGCACATACTGGATGATTCCTGAGCGCAATGATTGAATAGTCAA |
| CPS1t_R | | CTCATCCCGACCTTCCATTATTTGACACTTGATTTGACACTTCT |
| YPRC*delta*15_RF | | AATGGAAGGTCGGGATGAG |
| YPRC*delta*15_RR | | *GTTTAAAC*TTATTAAAGCTTGATAAATTACTGAA |
| **PCR verification of P*_GAL1_-INO2-*T*_CPS1_*, *YPRCdelta15* homologous arm with *HphMX* marker** | | |
| YPRC*delta*15_VF | | GCAGCCGCTACCAAACAG |
| YPRC*delta*15_VF | | ACTATCCGATAACGCCAGGC |
| **For construction of Δ*gal80::HIS3*** | | |
| gal80_F | | aatctcgatagttggtttcccgttctttccactcccgtcatgCGTTTTAAGAGCTTGGTGAGC |
| gal80_R | | ttcgctgcactgggggccaagcacagggcaagatgcttttaCCTGATGCGGTATTTTCTCC |
| **PCR verification of Δ*gal80*** | | |
| gal80_VF | | CATCACATATCACTGCTGGTCCT |
| gal80_VR | | TGAAGCTATGATGGAAGGATGC |
| **For construction of Δ*gal1* Δ*gal7* Δ*gal10::HIS3*** | | |
| gal1710_F | | AGAAAAAATATGATATGAATGAATATTCCACTTTCTTTCGTTTTAAGAGCTTGGTGAGC |
| gal1710_R | | GAGAAGTTGTTCTGAACAAAGTAAAAAAAAGAAGTATACCCTGATGCGGTATTTTCTCC |
| **PCR verification of Δ*gal1* Δ*gal7* Δ*gal10*** | | |
| gal1710_VF | | AAACTTCAACAGAGCCTAAAATTTGA |
| gal1710_VR | | AAACGCAGCGGTTGAAAGC |
| **For construction of Δ*ypl062w::KanMX*** | | |
| ypl062w_F | | ACTGCCGTCACATACGACACTGCCCCTCACGTAAGGGCTCTGTTTAGCTTGCCTCGTCC |
| ypl062w_R | | CACCCCGAATTTATTACGAATTTGCCCACATGGTCGGTGGTTTTCGACACTGGATGGCG |
| **PCR verification of Δ*ypl062*** | | |
| ypl062w_VF | | ACTGCACCTCCTAACATCACCA |
| ypl062w_VR | | TCAGCAGTGTCAAAGTGTAGCTTAG |
| **For construction of Δ*yjl064w::BleMX*** | | |
| yjl064w_F | | GTTCACCACATAGGCGGAGTAAACTTCATTAGGGGGCTCTGTTTAGCTTGCCTCGTCC |
| yjl064w_R | | ACAAGAGAGAATAGCGTCAGGATAGCTCGCTCGATGTGAGTTTTCGACACTGGATGGCG |
| **PCR verification of Δ*yjl064w*** | | |
| yjl064w_VF | | GTCCTTTTCGTTTGTAACCTCCT |
| yjl064w_VR | | GCGCTAACAATGTGCGTATTC |
| **For construction of Δ*rox1::BleMX*** | | |
| rox1_F | | ACTTCTTCACACAAAAGAACGCAGTTAGACAATCAACATCTGTTTAGCTTGCCTCGTCC |
| rox1_R | | ATATAGTATAATATAATATAACGGAAAGAAGAAATGGAAGTTTTCGACACTGGATGGCG |
| **PCR verification of Δ*rox1*** | | |
| rox1_VF | | TTCCGGTTGTAAGCCCTTCCACTT |
| rox1_VR | | CTGCTCTATCTTATTTGCTAATTGTAGTT |
| **For construction of Δ*dos2::BleMX/HphMX*** | | |
| dos2_F | | TTTCCTTTCTACCATAATACGTCATGAAAAGTATGTAATCTGTTTAGCTTGCCTCGTCC |
| dos2_R | | CAACAAAAAAAGAACACAACATAAGGGTACAGCTAGTGAGTTTTCGACACTGGATGGCG |
| **PCR verification of Δ*dos2*** | | |
| dos2_VF | | TGTTCTATTTCCACCCACAGC |
| dos2_VR | | CATAGCAATTGTGAACCCATAGT |

Homologous overhang-nucleotides were underlined and the restriction site *Pme*I was in italic.

**Table S2.** Distant genetic loci involved in this study

| **Gene name** | **Description** | **Reference** |
| --- | --- | --- |
| *YPL062W* | a dubious open reading frame , whose mutant has lower levels of glycogen | [1] |
| *ROX1* | a heme-dependent transcriptional repressor for enzymes in mevalonate pathway and ergosterol synthesis | [2] |
| *YJL064W* | a dubious open reading frame | [3] |
| *DOS2* | an uncharacterized protein involved in miconazole resistance | [4] |
| *INO2* | a transcription factor involved in phospholipid biosynthesis | [5] |

**
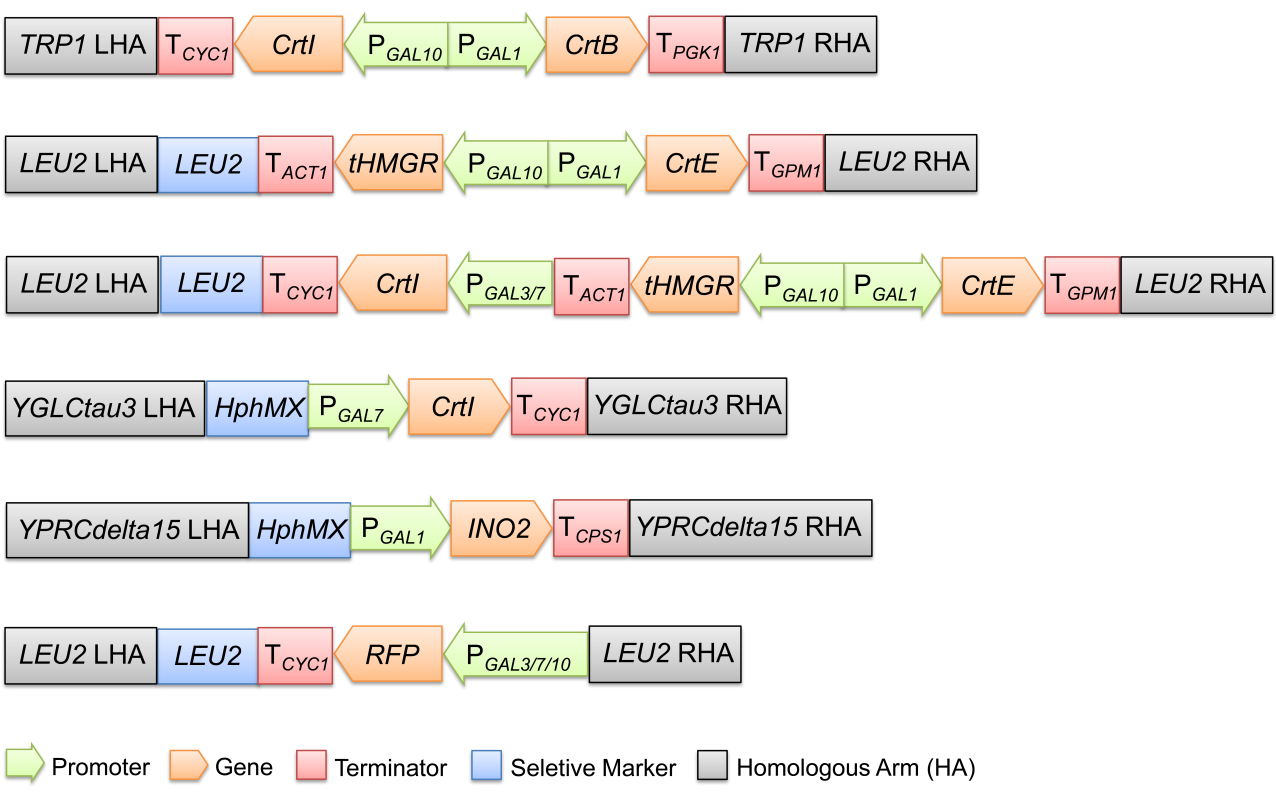
**

**Figure S1.** Integration modules constructed in this study. LHA and RHA represent left homologous arm and right homologous arm, respectively.


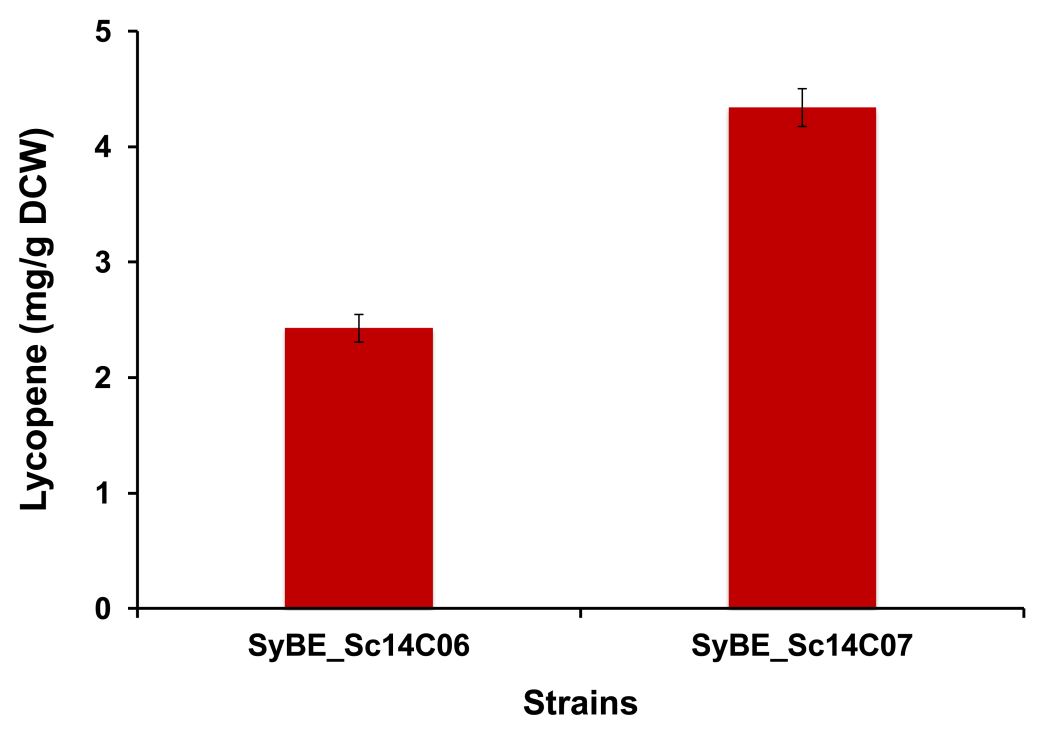


**Figure S2.** Comparison of Δ*gal1* Δ*gal7* Δ*gal10* strain and Δ*gal80* strain on lycopene production. Production of lycopene in shake-flask cultures of SyBE_Sc14C06 (Δ*gal80*) and SyBE_Sc14C07 (Δ*gal1* Δ*gal7* Δ*gal10*) in YPD medium containing no galactose and 10 g/L galactose, respectively. The error bars represent standard deviation calculated from triplicate experiments.


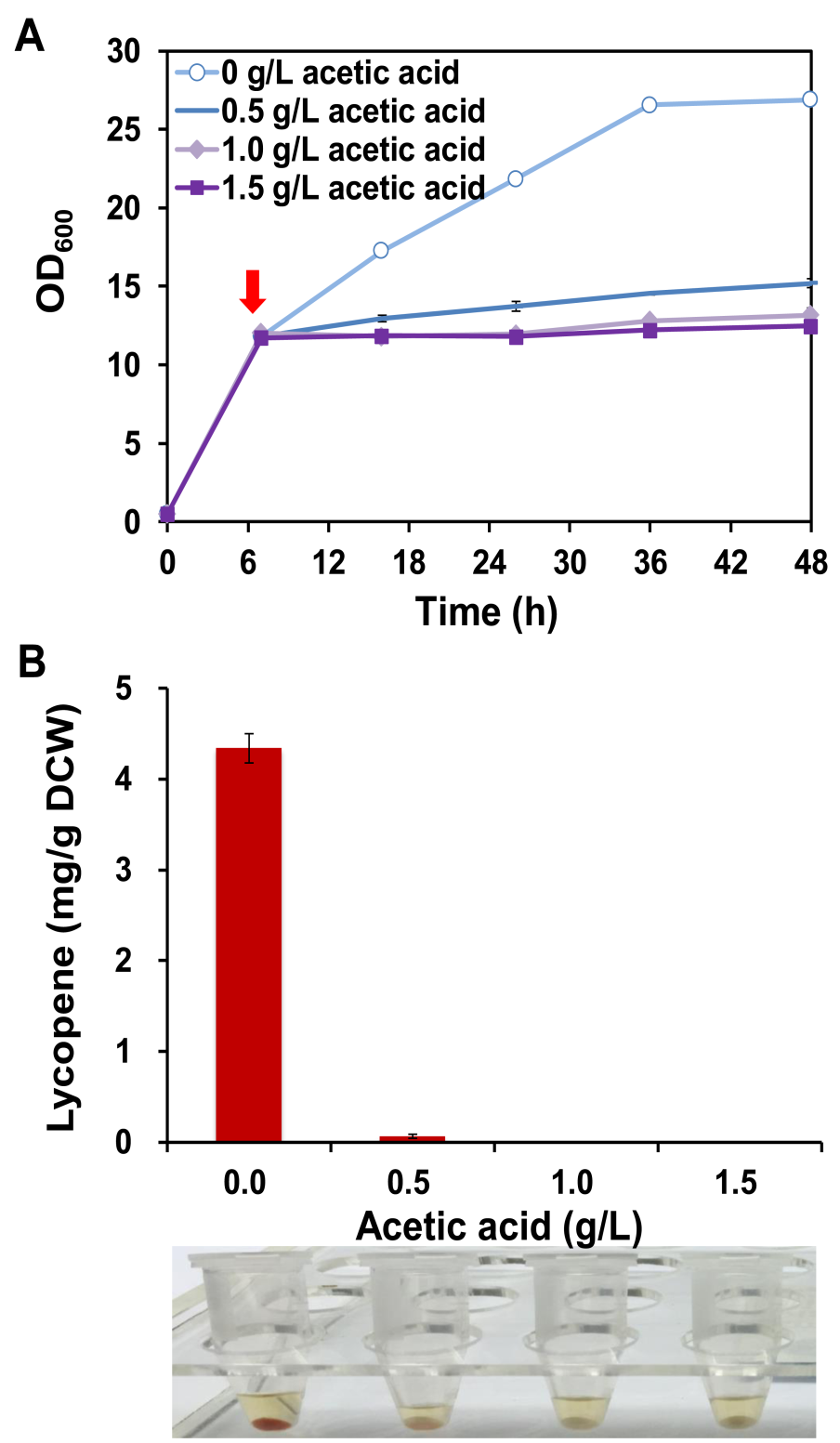


**Figure S3.** The effects of acetic acid addition on cell growth (A) and lycopene production (B) in strain SyBE_Sc14C07. Different amounts of acetic acid were added into YPDG media after 7h of shake-flask cultivations at a final concentration of 0-1.5 g/L. The arrow indicated the time of 7 h for acetic acid addition when the glucose was exhausted. The error bars represent standard deviation calculated from triplicate experiments.


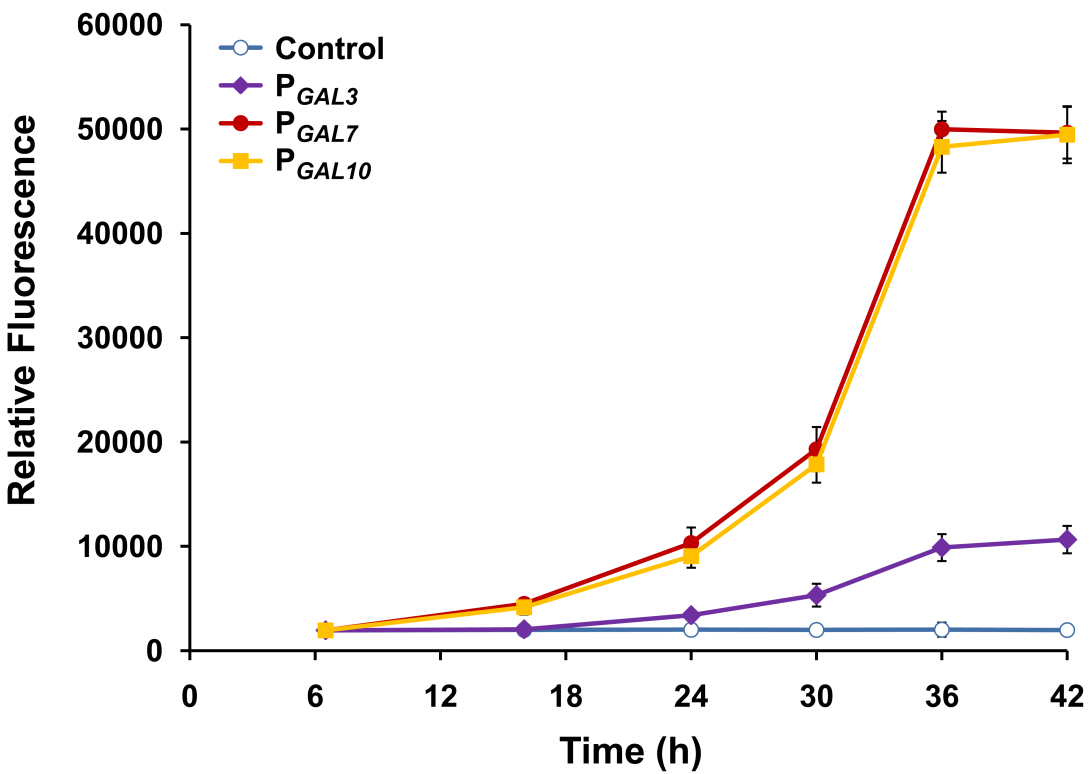


**Figure S4.** Time course of promoter strengths. Strengths of P*_GAL3_*, P*_GAL7_* and P*_GAL10_* were characterized in *S. cerevisiae* SyBE_Sc14C10 background by relative fluorescence intensity of red fluorescent protein (RFP). The strain with no promoter fused with RFP (SyBE_Sc14C40) was used as the control. The relative fluorescence was the ratio of the fluorescence to OD_600_ for each strain (SyBE_Sc14C40-SyBE_Sc14C43). The error bars represent standard deviation calculated from triplicate experiments.


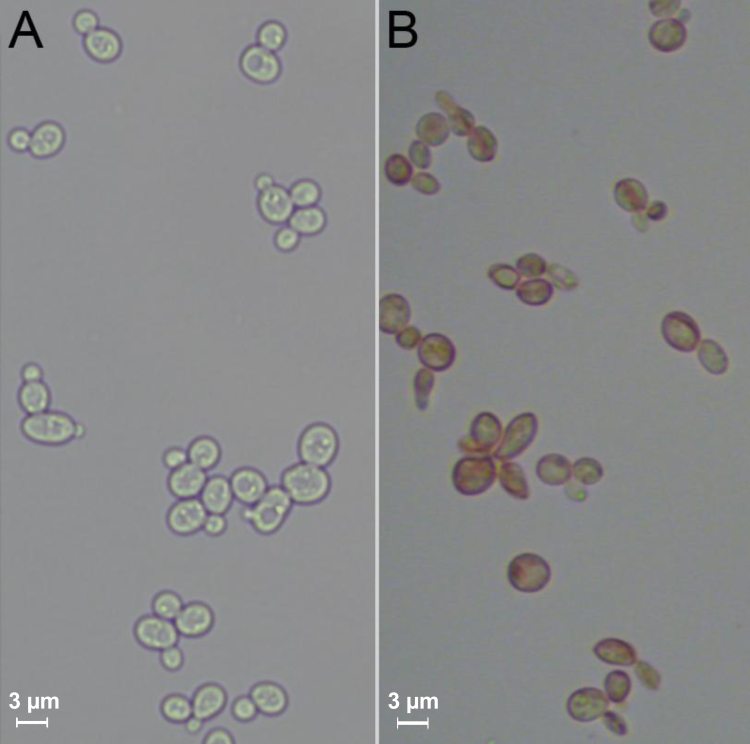


**Figure S5.** Microscopic images of lycopene-producing strain. Strain SyBE_Sc14C45 was imaged at 36 h of shake-flask cultivations in YPD medium (A) and YPDG medium (B), respectively.


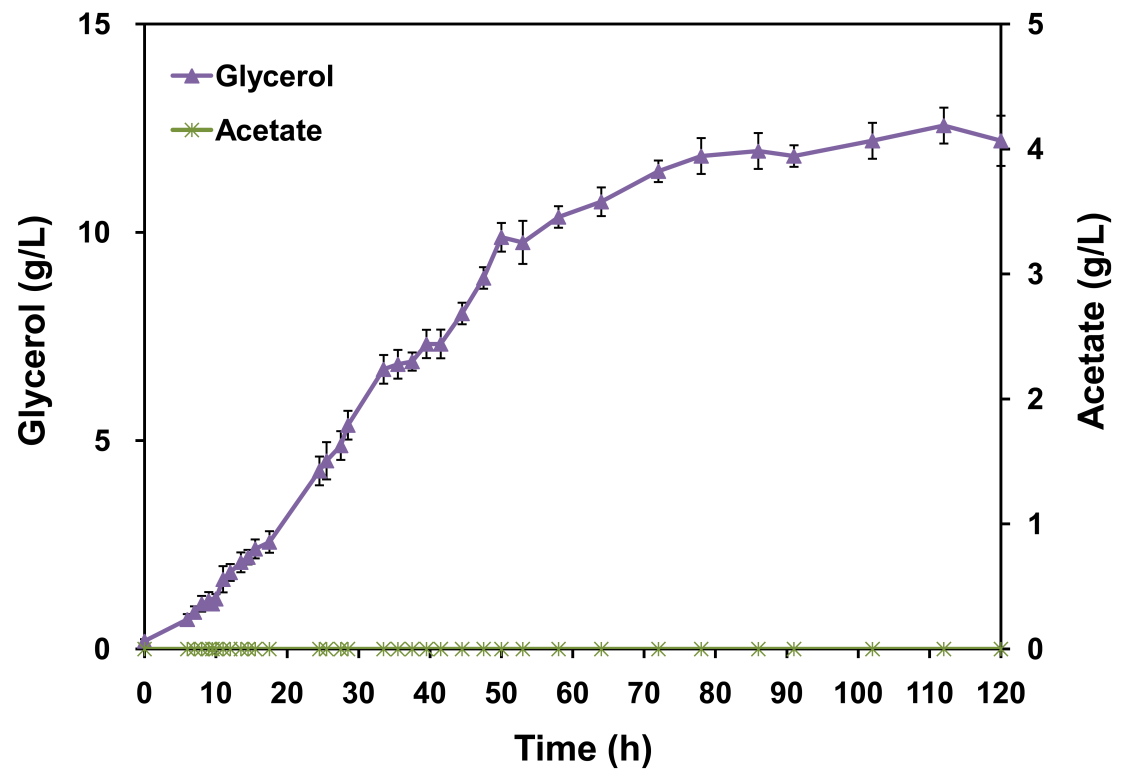


**Figure S6.** Profile of glycerol and acetate accumulation. Glycerol and acetate concentrations of strain SyBE_Sc14D14 were monitored during fed-batch fermentation in 5-L bioreactors. The error bars represent standard deviation calculated from duplicate experiments.

**Figure S7.** Sequences of codon-optimized genes.

> *CrtE* from *Pantoea agglomerans*

atggtttctggttctaaggctggtgtctcaccacacagggagattgaggtcatgaggcagtctattgacgatcacttggctggtttgttgcctgagactgactctcaggacattgtctcattggcaatgagggagggtgtcatggctccaggtaagaggataaggcctttgttgatgttgttggcagctagggacttgaggtaccagggttctatgcctactttgttggacttggcttgcgctgtcgaattgactcacactgcatcattgatgttggacgacatgccttgcatggacaacgcagaattaaggaggggtcagccaacaacacacaagaagttcggtgagtcagtcgcaattttggcatcagttggtttgttatcaaaggctttcggattgattgctgcaactggtgacttaccaggtgagaggagggcacaggctgtcaacgagttgtctactgctgtcggagtccagggattggtcttgggtcagttcagggacttgaacgacgcagctttggacaggactccagacgctatattgtctacaaaccacttaaagacaggaattttgttctctgctatgttgcagatagtcgctattgcatctgcttcttctccatctacaagggagactttgcacgctttcgcattggacttcggtcaggctttccagttgttggacgacttgagggacgatcacccagagacaggaaaggacaggaacaaggatgcaggtaaatcaactttggtcaacaggttaggtgcagacgctgctaggcagaagttaagggagcacattgactctgctgacaagcacttgactttcgcttgcccacagggaggtgctattaggcagttcatgcacttgtggttcggtcaccacttagctgactggtcacctgtcatgaagatagcttaa

> *CrtE* from *Sulfolobus acidocaldarius*

atgtcttactttgataattactttaatgaaattgttaattctgttaacgacattattaaatcttatatttctggtgatgttccaaaattatatgaagcttcttatcatttgtttacatctggtggtaaaaggctgaggccgttaatattgacaatttcttctgacttgttcggtggtcaaagggagagagcttactacgcaggtgctgctattgaggtcttgcatacttttactttagttcacgatgatattatggatcaagataatattagaaggggtttgcctactgtccacgtcaagtacggtttgcctttggctatattggctggtgatttgttgcatgctaaggcattccagttgttgacacaggctttgaggggtttgccatcagaaacaataattaaagctttcgatatttttactagatctattattattatttctgagggtcaggctgtcgacatggaatttgaagatagaattgatattaaggaacaagagtatttggatatgatttctagaaagactgctgctttgttttctgcttcttcttctattggagcattgattgctggtgctaatgacaatgatgttagattgatgtctgattttggtactaatttgggtattgcttttcagatagttgacgatattttgggattgacagctgacgagaaggagttgggtaagcctgtcttctctgacattagggagggtaaaaagactattttggtcattaaaactttagaattgtgtaaggaagatgagaagaagattgttttaaaagctttaggtaacaaatctgcttctaaggaagaattgatgtcatctgctgatattattaagaaatattcattagattacgcttacaatttggctgaaaaatactataagaatgctattgattcattgaaccaagtttcttctaagtcagatattcctggtaaggctttgaaatatttagctgaatttacaattagaagaaggaagtaa

> *CrtE* from *Archaeoglobus fulgidus*

atgttgaaagaagaaattgctaaaagagctgaaattattaataaagctattgaagaattgttgccagagagggagccaattggtttgtacaaggctgctaggcacttgattaaggctggtggtaagaggctgaggccggttatttctttgttggctgtcgaagctttgggtaaggattatagaaaaattattccggcggcggtttctattgaaactatacataatttcactttggttcatgatgatataatggatagagatgaaatgaggaggggtgtccctactgttcacagggtctacggtgaggcaactgctattttggctggtgacactttgtttgctgaagcttttaagttgttgacaaaatgtgatgttgaatctgaaggtattagaaaggctacagaaatgttatctgatgtttgcattaaaatttgtgaaggtcaatactatgatatgtcatttgagaaaaaagaatcagtttcagaagaagagtatttgagaatggttgaattgaaaacaggtgtcttgattgctgcttcagctgctttgccagctgtcttgttcggtgaatctgaagaaattgtcaaagctttatgggactacggtgtcttgtctggaattggttttcaaattcaagatgatttgttagatttgacagaagaaactggtaaagattggggttctgatttgttgaagggtaagaaaactttgattgttataaaagcttttgaaaaaggtgttaaattgaaaacttttggtaaagaaaaggctgatgtttctgaaattagagacgatattgaaaagttgagagaatgcggtgctattgactacgctgcatctatggctaggaagatggctgaggaggctaagagaaagttggaagttttgcctgaatctaaagctaaagaaacattgttagaattgacagattttttagttactagaaagaaataa

> *CrtE* from *Blakeslea trispora*

atgttgacttcttctaaatctattgaatcttttccaaaaaatgttcaaccatatggtaagcattatcaaaatggtttggagccagtcggtaagtctcaggaggacattttgttagagccattccactacttgtgctctaaccctggtaaggacgtcaggactaagatgatagaagcattcaatgcatggttgaaggtcccaaaggacgatttgatagttattacaagggttattgagatgttgcactctgcatcattattgattgacgacgtcgaggacgactcagtcttgaggagaggtgtccctgctgcacaccacatttacggaacaccacagactataaactgtgctaattacgtctacttcttggctttaaaggagattgctaagttaaacaaacctaacatgattactatttacacagacgagttgattaacttgcacaggggtcagggtatggagttgttctggagggacacattgacttgcccaacagagaaggagttcttggacatggtcaatgacaaaactggtggattgttgaggttggctgtcaagttgatgcaggaggcttctcagtcaggaactgactacacaggattggtttcaaagattggaatacatttccaggtcagggacgattatatgaatttacagtcaaaaaactacgcagacaacaagggattctgcgaggacttgacagagggaaagttctctttcccaataatacactcaattaggtctgacccatcaaacaggcagttgttgaacatattgaaacagagatcttcatctattgaattgaaacaattcgcattgcagttattggaaaatacaaacactttccaatattgcagagacttcttgagggtcttggagaaggaggcaagagaggaaattaaattgttgggtggtaatataatgttagaaaagattatggacgtcttgtctgtcaacgaataa

> *CrtE* from *Taxus x media*

atggcttataccgcaatggcagcaggaactcagtcattgcagttgaggacagtcgcctcttaccaggagtgcaactcaatgaggtcttgcttcaagttgaccccattcaagtcattccacggtgtcaacttcaacgttccttctttaggtgccgccaactgcgaaatcatgggtcacttgaaattgggttctttgccatacaaacagtgttcagtatcatctaagtcaactaagactatggcccagttggtagatttggcagagaccgagaaagccgagggaaaggatatcgagttcgattttaacgagtatatgaagtctaaggctgtcgctgttgatgcagccttggataaggccatccctttggagtatccagagaagatccatgagtctatgaggtactcattgttggccggaggaaaaagggtcagacctgcattatgcatcgctgcttgcgagttagtaggtggttctcaggacttggccatgccaaccgcatgtgccatggaaatgattcataccatgtcattgattcacgatgatttgccttgcatggacaacgacgacttcagaaggggaaagcctaccaatcacaaggttttcggagaggacactgctgttttagccggtgacgcattgttatctttcgcttttgaacacatcgccgttgccacatcaaaaactgtcccatctgacaggaccttgagagtcatttctgagttgggtaaaaccatcggttcacagggattggtcggaggtcaggtagtcgacatcacttctgagggagacgccaacgtcgacttaaagacattggagtggattcacattcacaagactgccgtcttgttggaatgctctgttgtttctggaggaatcttgggtggagctaccgaggatgagattgctagaataagaagatacgccaggtgcgtcggtttgttgttccaggttgtcgacgacattttggatgtcaccaagtcttcagaggaattgggaaagaccgccggtaaagacttattgaccgacaaggctacctaccctaagttgatgggtttggagaaggccaaagagtttgcagcagaattagctaccagggcaaaggaagagttgtcatcattcgaccagatcaaggcagcccctttgttaggattggccgattacatcgctttcaggcaaaactaa

> *CrtB* from *Paracoccus* sp.

atgtccgacttagttttgacctctactgaagccataacacaaggttcccaatctttcgctactgctgccaagttgatgcctcctggtatcagagatgacacagtcatgttgtatgcctggtgtagacatgctgatgacgtaattgatggtcaagcattaggttccagacctgaagctgttaatgatccacaagcaagattggatggtttaagagctgacactttggctgcattacaaggtgacggtcctgtaacaccaccttttgccgctttgagagccgttgctagaagacatgatttccctcaagcatggccaatggacttaattgaaggttttgcaatggatgttgaagccagagactacagaaccttggatgacgttttagaatattcttaccacgtcgccggtatagttggtgtcatgatggctagagtaatgggtgttagagatgaccctgttttggatagagcctgtgacttgggtttagctttccaattaaccaacattgcaagagatgttatagatgacgccagaatcggtagatgctatttgcctggtgactggttagaccaagcaggtgccagagtcgatggtccagtaccttcaccagaattgtacactgttattttgagattgttggatgcagccgaattgtattacgcttccgcaagagtcggtttggctgatttgccacctagatgcgcatggagtatagctgcagccttgagaatctatagagcaatcggtttgagaatcagaaagggtggtccagaagcatatagacaaagaatctctacttcaaaagctgcaaagattggtttgttaggtataggtggttgggatgttgctagatcaagattaccaggtgccggtgtatcaagacaaggtttatggacaagaccacatcacgcctaa

> *CrtB* from *Pantoea agglomerans*

atgtcacaaccaccattattggaccacgctacacaaactatggcaaacggttctaaatctttcgctactgctgctaaattattcgacccagcaacaagaagatctgtattgatgttgtacacctggtgtagacattgcgatgacgttatagatgaccaaactcacggttttgcttcagaagctgcagccgaagaagaagctacacaaagattggcaagattaagaactttgacattagctgcattcgaaggtgccgaaatgcaagatccagcttttgccgctttccaagaagttgcattaacccatggtattactcctagaatggctttggatcacttagacggttttgcaatggatgtcgcccaaacaagatacgtaaccttcgaagacactttaagatattgttaccatgtcgccggtgttgtcggtttgatgatggctagagtaatgggtgttagagatgaaagagttttagatagagcatgtgacttgggtttagccttccaattgacaaacatagctagagatataatagatgacgcagccatagacagatgctatttgccagctgaatggttacaagatgcaggtttgactcctgaaaattacgctgcaagagaaaacagagccgctttagccagagttgctgaaagattgatagatgcagccgaaccatattacatctcttcacaagctggtttgcatgatttgccacctagatgcgcatgggccattgctaccgcaagatctgtttacagagaaatcggtattaaagtcaaggctgcaggtggttccgcatgggatagaagacaacacacttctaaaggtgaaaagatcgctatgttgatggccgctcctggtcaagttattagagcaaagaccaccagagtcaccccaagaccagccggtttatggcaaagacctgtttaa

> *CrtI* from *Paracoccus* sp.

atgaacgctcactcccctgccgcaaagactgccatagttatcggtgccggtttcggtggtttagccttagccattagattacaaagtgccggtattgctactacattggtagaagctagagataagccaggtggtagagcatacgtttggcatgatcaaggtcacttatttgacgcaggtccaacagttataaccgatcctgacgcattgaaagaattatgggccttgaccggtcaagatatggctagagacgtaactttgatgccagtttctcctttttatagattaatgtggccaggtggtaaagttttcgattacgtcaatgaagccgaccaattggaaagacaaatagctcaattcaatcctgatgacttagaaggttatagaagattcagagattacgctgaagaagtttatcaagaaggttacgtcaagttgggtacagtaccattcttgaagttaggtcaaatgttaaaagctgcacctgctttaatgaaattggaagcatacaagtcagtacatgcaaaggttgccacttttattaaggatccatatttgagacaagcattctcttaccacacattgttagtaggtggtaatcctttttcaacctcttcaatctatgcattgatacatgccttggaaagaagaggtggtgtttggttcgctaagggtggtacaaatcaattagtcgctggtatggtagcattatttgaaagattgggtggtcaaatgttgttaaacgctaaagttgcaagaattgataccgacggtccaagagccactggtgtcacattggctgatggtagagccttaactgctgacatggttgcatccaatggtgacgtcatgcataactatagagacttgttaggtcacacagctagaggtcaatccagagccaagagtttgaatgctaaaagatggtccatgagtttatttgtcttgcatttcggtttaagagaagcaccaaaggatgtagcccatcacactatcttgttcggtcctagatacaaggaattagttaacgaaatctttaaaggtccaaagttggctgaagatttctccttgtacttacatagtccatgtaccactgaccctgaaatggcaccacctggcatgtccactcattatgttttggccccagtccctcacttaggtagagctgatattgactgggcagttgaaggtccaagatacgccgatagaatcttagctagtttggaagaaagattgatcccaaatttgagagctaacttaacaaccactagaatttttacaccttctgatttcgcatcagaattgaatgcccatcacggttctgctttttcagtcgaaccaattttaacccaatcagcttggttcagacctcataacagagataagactatcagaaacttctatttggttggtgcaggtacacacccaggtgccggtatccctggtgtcgtcggttccgctaaggctacagcacaagttatgttatctgatttggcttccgcataa

> *CrtI* from *Pantoea agglomerans*

atgaagaagacagtcgtcataggagcaggattcggtggtttggctttggcaattaggttacaggcagctggaataccaacagtcttgttggagcagagggacaagccaggaggtagggcatacgtctggcacgaccagggttttacattcgacgcaggaccaactgttataactgaccctacagcattggaggcattgttcactttggctggtagaaggatggaggactatgtcaggttgttgccagtcaagcctttctacaggttgtgctgggagtcaggtaagacattggactacgctaacgactcagctgagttggaggctcaaattacacaattcaacccaagggacgtcgagggttacaggaggttcttggcatactcacaagcagtttttcaggagggatacttgagattgggatctgtcccattcttgtcttttagggatatgttgagggcaggtccacagttgttgaagttgcaggcttggcagtctgtctaccagtcagtctcaaggttcattgaagacgagcacttaaggcaggctttctcattccattcattgttagttggtggaaatcctttcactacttcatctatttatacattaatacacgctttggaaagagagtggggtgtctggttccctgagggtggtactggagctttggtcaacggaatggttaagttgttcactgacttgggaggagaaatagagttgaacgcaagggtcgaagagttggttgtcgcagacaacagggtctcacaggtcagattagctgacggaagaattttcgacacagacgctgtcgcttctaacgcagacgtcgttaacacttataaaaagttgttgggtcatcaccctgtcggacagaagagggctgctgcattggagagaaagtcaatgtcaaattctttatttgttttatatttcggtttaaaccagccacactctcagttggcacaccacactatttgctttggaccaagatacagagagttgatagacgagatatttactggatcagcattggcagatgacttttcattgtacttgcactctccatgcgtcacagacccatcattggctccaccaggttgcgcatcattctacgtcttggctccagtccctcacttgggaaacgcaccattggattgggcacaggagggaccaaagttaagggatagaatttttgattatttagaggaaaggtacatgcctggtttgagatcacaattggtcactcagaggatttttacacctgctgacttccacgacacattggacgctcacttgggttctgctttctctattgagccattgttgacacagtcagcttggttcaggcctcacaacagggactctgacatagctaacttgtatttggtcggtgctggtacacaccctggtgcaggtattcctggtgtcgttgcttctgcaaaggcaacagcatctttgatgatagaggacttgcagtaa

> *CrtI* from *Blakeslea trispora*

atgtctgatcagaagaagcacattgtcgtcataggtgctggaataggaggtactgcaacagcagcaaggttagcaagggagggtttcagagtcactgtcgtcgagaagaacgacttctctggaggaaggtgctctttcattcaccacgacggtcacaggttcgaccagggaccttcattgtacttgatgcctaagttgtttgaggacgctttcgctgacttagacgagaggataggagaccacttggacttattaagatgtgacaacaattacaaagtccatttcgacgacggtgacgctgtccaattgtcatcagacttaacaaagatgaagggtgagttggacaggattgagggacctttaggattcggtaggttcttagatttcatgaaagagacacacgtccactacgagcagggtacattcattgctataaagagaaacttcgaaactatatgggacttaataaggttacagtacgtcccagagatttttaggttgcacttattcggtaagatatacgacagagcatcaaaatacttccaaacaaaaaagatgaggatggcttttacttttcaaacaatgtacatgggtatgtcaccttacgacgcacctgcagtctactcattgttgcaatatacagagttcgcagagggaatttggtacccaaggggtggtttcaacatggtcgtccaaaagttggagtctatagcttctaagaagtacggagctgagttcaggtaccaatctcctgtcgctaagattaacactgtcgataaagacaagagggtcactggtgtcactttggagtctggagaagtcattgaggcagacgctgtcgtctgcaacgctgacttggtctacgcttaccaccacttgttgccaccttgcaactggacaaagaagactttggcatctaagaaattaacatcttcatcaatttctttttactggtcaatgtctactaaggtccctcaattggacgtccacaacattttcttggctgaggcttacaaggagtcattcgacgagatttttaacgatttcggtttgccttctgaagcatctttctacgtcaacgttccttcaaggatagacgagtctgcagcacctccaaataaggactcaattatagttttagttccaattggtcacatgaagtctaagacaggtaactcagcagaggagaactacccagagttggtcaacagggctagaaagatggtcttggaggtcatagagaggaggttgggagtcaacaacttcgctaacttgatagaacacgaggaggtcaacgacccatcagtctggcaatctaagttcaacttgtggaggggatcaatattaggtttatcacatgatgtctttcaggttttgtggttcagaccttcaacaaaggactctactaacagatatgacaatttatttttcgtcggtgcatcaactcaccctggtacaggagtcccaatagtcttggcaggatctaaattaacttctgaccaggtctgtaagtcattcggacaaaaccctttgcctaggaagttacaggactctcagaagaaatatgcacctgagcaaacaaggaagactgagtcacactggatttattactgcttagcatgctactttgtcactttcttgttcttctatttctttcctagggacgacactactactccagcatcttttattaatcagttgttgccaaacgtcttccaaggacagaactctaacgatattaggatataa

**References**

1. Fisk DG, Ball CA, Dolinski K, Engel SR, Hong EL, Issel-Tarver L, Schwartz K, Sethuraman A, Botstein D, Cherry JM. *Saccharomyces cerevisiae* S288C genome annotation: a working hypothesis. Yeast. 2006;23(12):857-65.

2. Francois IE, Bink A, Vandercappellen J, Ayscough KR, Toulmay A, Schneiter R, van Gyseghem E, Van den Mooter G, Borgers M, Vandenbosch D, et al. Membrane rafts are involved in intracellular miconazole accumulation in yeast cells. J Biol Chem. 2009;284(47):32680-5.

3. Giaever G, Chu AM, Ni L, Connelly C, Riles L, Veronneau S, Dow S, Lucau-Danila A, Anderson K, Andre B, et al. Functional profiling of the *Saccharomyces cerevisiae* genome. Nature. 2002;418(6896):387-91.

4. Henry KW, Nickels JT, Edlind TD. ROX1 and ERG Regulation in *Saccharomyces cerevisiae*: Implications for Antifungal Susceptibility. Eukaryot Cell. 2002;1(6):1041-4.

5. Nikoloff DM, Henry SA. Functional characterization of the INO2 gene of *Saccharomyces cerevisiae*. A positive regulator of phospholipid biosynthesis. J Biol Chem. 1994;269(10):7402-11.
